# Identification of Surrogate Biomarkers for Mucopolysaccharidosis Type IVA

**DOI:** 10.3390/ijms26104940

**Published:** 2025-05-21

**Authors:** Yasuhiko Ago, Shaukat Khan, Kimberly Klipner, Allison Bradford, Shunji Tomatsu

**Affiliations:** 1 Nemours Children’s Health, Wilmington, DE 19803, USA; yasuhiko.ago@nemours.org (Y.A.); shaukat.khan@nemours.org (S.K.); kimberly.klipner@nemours.org (K.K.); allison.bradford@nemours.org (A.B.); 2 Faculty of Arts and Sciences, University of Delaware, Newark, DE 19716, USA; 3 Department of Pediatrics, Graduate School of Medicine, Gifu University, Gifu 501-1193, Japan; 4 Department of Pediatrics, Thomas Jefferson University, Philadelphia, PA 19107, USA

**Keywords:** mucopolysaccharidosis IVA, NT-proCNP, keratan sulfate, biomarkers, enzyme replacement therapy, skeletal dysplasia

## Abstract

Mucopolysaccharidosis type IVA (MPS IVA, Morquio A syndrome) is a rare inherited disorder characterized by skeletal dysplasia due to deficient N-acetylgalactosamine-6-sulfate sulfatase activity, resulting in glycosaminoglycan (GAG) accumulation. Identifying accurate biomarkers reflecting clinical severity and therapeutic response remains challenging. This study evaluated potential surrogate biomarkers, including N-terminal pro-C-type natriuretic peptide (NT-proCNP), collagen types I and II, mono-sulfated keratan sulfate (KS), di-sulfated KS, and chondroitin-6-sulfate (C6S), in blood and urine samples from 60 patients ranging from 1 to 62 years of age. NT-proCNP levels were significantly elevated in patients of all ages and negatively correlated with growth impairment, especially after 8 years of age. Collagen type I levels significantly increased in adult patients, whereas collagen type II showed age-dependent elevations. Urinary KS, in mono- and di-sulfated forms, demonstrated moderate negative correlations with growth impairment. Moreover, NT-proCNP, mono- and di-sulfated KS in plasma, and urinary di-sulfated KS were not affected by enzyme replacement therapy in patients younger than 12 years, unlike urinary mono-sulfated KS. In conclusion, NT-proCNP has emerged as a promising independent biomarker reflecting the severity of skeletal dysplasia and possibly the near-future growth rate. These findings highlight the potential role of NT-proCNP in clinical assessment and monitoring therapeutic efficacy, addressing current unmet needs in MPS IVA management.

## 1. Introduction

Mucopolysaccharidoses (MPSs) are a group of inherited lysosomal storage disorders caused by deficiencies in enzymes required to degrade glycosaminoglycans (GAGs), which are integral to connective tissue. The inability to break down GAGs results in accumulation in multiple tissues and organs, leading to systemic dysfunction. Depending on the specific enzyme deficiency, MPSs are classified into several subtypes [1]. In some instances, a phenomenon known as pseudodeficiency can potentially complicate diagnosis and prognosis, highlighting the importance of biomarkers that accurately reflect the stage and prognosis of the disease [2].

Among these, MPS IVA (Morquio A syndrome) is characterized by a deficiency in the N-acetylgalactosamine-6-sulfate sulfatase (GALNS) enzyme, which leads to the accumulation of keratan sulfate (KS) and chondroitin-6-sulfate (C6S) [3]. MPS IVA is a rare genetic disorder that causes growth impairment and skeletal abnormalities with an estimated incidence of 0.11 to 1.32 per 100,000 live births, depending on the region [4,5]. The resultant skeletal dysplasia is primarily driven by the accumulation of GAGs (KS and C6S), which disrupts the function of chondrocytes in the growth plates, impairs cartilage matrix development, and inhibits bone matrix mineralization [6]. The severity of bone and cartilage damage differs by the severity of the phenotype (attenuated or severe) and the type of bone affected [7]. Bone growth by endochondral ossification is affected more severely than other types of MPS. Thus, it is a type of skeletal dysplasia affecting bone and cartilage growth. The hallmark feature of MPS IVA is progressive and characterized by incomplete or defective endochondral ossification and a successive imbalance of growth [3,8,9,10]. The skeletal-related symptoms of MPS IVA include prominent forehead, abnormal face with a large mandible, disproportionate short-trunk dwarfism with short neck, cervical spine instability with odontoid hypoplasia, cervical spinal cord compression, pectus carinatum, tracheal deviation and obstruction, restrictive lung, flaring of the rib cage, kyphoscoliosis, platyspondyly, hip dysplasia with coxa valga, genu valgum, hypermobile joints, waddling gait, and pes planus. The degree of imbalance in growth between bone and other organs and tissues contributes to the unique skeletal dysplasia and clinical severity, including the narrowing of the trachea. Respiratory failure has been shown as the primary cause of death in patients (63%), followed by cardiac failure (11%), post-traumatic organ failure (11%), complications of surgery (11%), and myocardial infarction (4%) [11].

Identifying therapeutic surrogate biomarkers for patients with MPS remains a significant challenge. Characterization of GAGs in urine, blood, and cerebrospinal fluid has been performed for several decades. The previous studies indicated no correlation between urine and serum GAG levels in MPS II and MPS IVA patients [12]. We found that blood and urine KS levels follow different age-dependent patterns and sulfation levels [13,14]. Another report in MPS I suggested that the oligosaccharide storage pattern (the ratio of heparan sulfate to dermatan sulfate) in urine reflects the storage in the kidney, which is different from the storage pattern in serum, liver, and brain [15]. These findings suggest that urinary GAG is derived primarily from GAGs stored or reabsorbed in the kidney. Some urinary GAG may be derived from small GAG fragments (i.e., oligosaccharides rather than a large saccharide chain) that filter through the kidneys from the circulation. Thus, blood GAGs would more closely reflect GAGs stored in the whole body and may be a more informative indicator of systemic disease burden than urinary GAGs.

In MPS IVA, blood and urinary KS levels correlate to some extent with clinical severity in the early and progressive stages of the disease, before the growth plate is destroyed, since KS synthesis decreases rapidly after the teenage years, and KS levels in MPS IVA patients are naturally normalized or subnormal by the age of 20 years [8,13,14,16,17,18,19]. Therefore, it can only be useful as a biomarker before the teenage years [8,20]. In addition, even before the teenage years, there was a lack of reliable biomarkers that correlate closely with skeletal symptoms, as described below.

Blood KS in MPS IVA directly indicates growth and/or repair of the cartilage, where it is mainly synthesized [20,21]. Most blood KS is derived from chondrocytes and their ECM; therefore, its reduction would be a more direct indicator of bone improvement. However, there is an overlap in blood KS levels between patients and age-matched controls [22,23]. On the other hand, urinary KS can distinguish more MPS IVA patients from age-matched controls than blood KS; however, there was no correlation between urinary KS reduction and clinical improvement of skeletal lesions during enzyme replacement therapy (ERT) [24,25]. After starting ERT, most patients showed a decrease in urinary KS levels because the enzyme is delivered to the kidney and digests KS stored in the kidney [26], but a concomitant decrease in blood KS levels has not yet been reported because urinary KS does not reflect blood KS coming directly from bone and cartilage, as explained above. Thus, urinary KS is functional in demonstrating the pharmacodynamic effects of therapy, but it does not provide a valuable surrogate biomarker of skeletal or clinical improvement during these therapies for MPS IVA [26]. In summary, blood KS reflects overall skeletal symptoms more accurately than urinary KS, particularly in patients undergoing ERT. However, it should be noted that blood KS is less sensitive than urinary KS when diagnosing patients. Conversely, urinary KS presents a challenge in effectively monitoring therapeutic response in patients receiving ERT.

Due to the current limitations described above, developing novel biomarkers remains an unmet challenge, prompting us to explore alternative biomarkers that can more accurately reflect the skeletal pathology of MPS IVA. In this study, we investigated N-terminal pro-C-type natriuretic peptide (NT-proCNP), collagen type I, and collagen type II as candidate biomarkers in human blood because of their importance in the skeletal system, particularly in the epiphyseal plate, connective tissues, and cartilage, respectively. Additionally, we separately evaluated two types of KS, mono-sulfated KS (Galβ1 → 4GlcNAc(6S)) and di-sulfated KS (Galβ1(6S) → 4GlcNAc(6S)), to elucidate their respective significance.

In addition to its role in the cardiovascular and neurological systems [27,28], C-type natriuretic peptide (CNP) plays a critical role in skeletal development [29]. CNP is synthesized in various tissues, including cartilage [30,31,32,33,34,35], and acts through the natriuretic peptide receptor B (NPR-B) on chondrocytes to activate the receptor’s guanylyl cyclase domain, increasing intracellular cGMP levels [36]. Elevated cGMP then activates protein kinase G (PKG), which phosphorylates various proteins controlling gene expression and metabolic processes. This phosphorylation influences transcription factors and other regulators, promoting chondrocyte maturation, extracellular matrix production, and balanced cell proliferation [36,37]. Through these coordinated intracellular events, CNP ensures the proper progression of chondrocyte differentiation and healthy growth plate development. As a result, the skeletal structure can expand and mature, underscoring CNP’s critical role in maintaining the equilibrium between chondrocyte growth and its progression to a fully differentiated state [36,38,39]. These processes are crucial for endochondral ossification in the growth plates, facilitating longitudinal bone growth. NT-proCNP, consisting of 50 amino acids, is the biologically inactive cleavage product of proCNP [40,41] and is secreted in equimolar amounts with CNP [42]. Based on the higher concentration of NT-proCNP in blood than that of CNP [43,44,45,46], NT-proCNP is thought to have a longer half-life than CNP in circulation, reflecting the systemic expression level of CNP better than CNP itself.

Collagen type I is the most abundant collagen in the human body, forming a critical structural component of bone, skin, tendons, and other connective tissues [47,48,49]. It provides the scaffold for bone mineralization and contributes to the strength and rigidity of the skeletal system [50,51,52]. Interestingly, studies have shown that the expression of mRNA encoding collagen type I increased in chondrocytes derived from MPS IVA patients [53,54].

Collagen type II, on the other hand, is predominantly found in cartilage [55] and serves as a marker for chondrocyte activity [56,57], especially in the proliferative zone in growth plates [58]. It is essential for maintaining the tensile strength and structural integrity of cartilage, including the growth plate [55,59]. As a critical component of the extracellular matrix, collagen type II supports the mechanical properties of cartilage and facilitates chondrocyte differentiation and matrix organization [60].

In this study, we focused on these three biomarkers (NT-proCNP, collagen types I and II) to elucidate their relationship with the skeletal manifestations of MPS IVA. By investigating these biomarkers with GAGs (C6S and KS) levels and genotyping, we aimed to identify a novel biomarker that can more accurately predict clinical severity, disease prognosis, and therapeutic efficacy relevant to improvements in bone lesions. Our findings have provided valuable insights into the pathophysiology of MPS IVA and informed the development of potential surrogate biomarkers.

## 2. Results

### 2.1. Subject Characteristics (Age, Sex, Race, Height)

**Figure 1:**
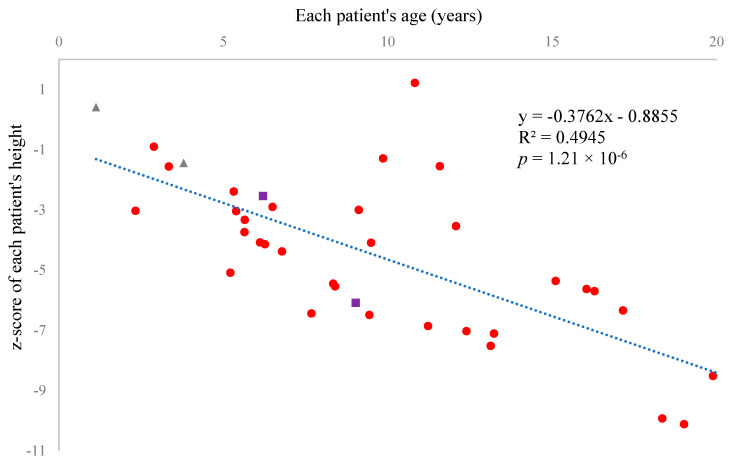
The Z-score of each patient’s height under 20 years showed a significant negative correlation with age. Each dot represents the z-score of height when each patient’s height was first recorded during the study period. Red round dots represent the patients who had received enzyme replacement therapy (ERT). Gray triangular dots represent the patients who had never received ERT. Purple square dots represent patients who had received hematopoietic stem cell transplantation (HSCT).

Sixty patients successfully provided us with blood and/or urine specimens. The characteristics of all participants are summarized in Table 1 (also in Table S1, including measured biomarkers). Thirty-three patients (55%) were female, and twenty-seven (45%) were male. Forty-four (73.3%) of the participants were White or Caucasian, five (8.3%) were Black or African American, three (5%) were South Asian, two (3.3%) were Southeast Asian, two (3.3%) were East Asian, and four (6.7%) were other/mixed race. The z-score of each patient’s height showed a significant negative correlation with age, indicating that current therapy is not effective enough to prevent growth impairment in MPS IVA (*r* = −0.703, *p =* 1.21 × 10^−6^, Figure 1).

### 2.2. Genotyping

Genetic testing of the *GALNS* gene revealed 8 homozygotes and 41 compound heterozygotes. In 10 patients, we could detect only one mutation on one allele. One patient could not be genetically tested since we could not obtain her genomic DNA (Table 1 and Table S1). Among the detected mutations, c.448delC (H150Tfs*3), c.633+1G>C, c.946G>A (G316R), and c.1339G>C (D447H) were not listed on the ClinVar website (https://www.ncbi.nlm.nih.gov/clinvar/ (accessed on 17 April 2025)), and we could not find any previously published articles describing these four mutations. The significance of the last two variants was predicted using online bioinformatics tools, MutationTaster (https://www.mutationtaster.org/index.html (accessed on 17 April 2025)) and PolyPhen-2 (http://genetics.bwh.harvard.edu/pph2/ (accessed on 17 April 2025)). Both variants were indicated as “disease-causing” and “probably damaging with a score of 1.000” by MutationTaster and PolyPhen-2, respectively. The remaining two variants were disease-causing mutations since one causes a frameshift that reduces the length of the entire peptide to less than a third of the original protein, and another one disrupts normal splicing by destroying the GT-AG rule [61], the basis of splicing.

### 2.3. Biomarkers (Collagen Type I, II, NT-ProCNP, and GAGs)—All Raw Data from the Patients Are Listed in Table S1

#### 2.3.1. NT-ProCNP

**Figure 2:**
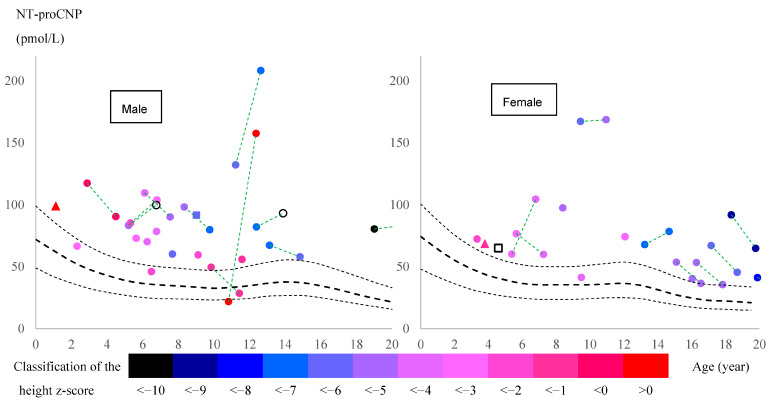
Correlation between age, NT-proCNP, and height of patients with MPS IVA. Males and females are shown in the left and right figures, respectively. Round dots represent patients treated with enzyme replacement therapy (ERT). Triangle markers represent patients who were never treated with ERT. Square markers represent patients treated with hematopoietic stem cell transplantation (HSCT). Filled markers represent patients for whom height at blood sample collection was recorded. Absence of a color indicates that the corresponding height dataset is missing. The color of each marker represents the z-score of each patient’s height calculated based on the CDC growth chart (https://www.cdc.gov/growthcharts/index.htm (accessed on 15 December 2024)). Black dashed lines show median, 5th and 95th percentiles for healthy controls [45]. When a patient has two data points, each data point is connected by a green dashed line.

Most pediatric patients, both males and females, had elevated levels above the 95th percentile for healthy controls of both sexes, as established by Olney et al. [45] (Figure 2). The increase was evident just before the age of pubertal growth spurt in healthy subjects. Focusing on patients with two data points at different ages before the age of peak pubertal growth spurt (all of whom had received ERT during these two periods), 5 out of 10 boys and 2 out of 3 girls showed an increase in NT-proCNP levels. Results from adults over 20 years are summarized in Figure S1, showing a significant difference between healthy controls and patients (*p =* 0.019).

A higher NT-proCNP level correlated with a lower z-score for height, indicating a severe growth impairment, especially between the ages of 8 and 13 years (17 subjects) (Figure 2). Including this age group, the partial correlation coefficient between the NT-proCNP level and the z-score of height was calculated, with age as a controlling factor, revealing a significant negative correlation in patients older than 8 years (Table 2).

**Figure 3:**
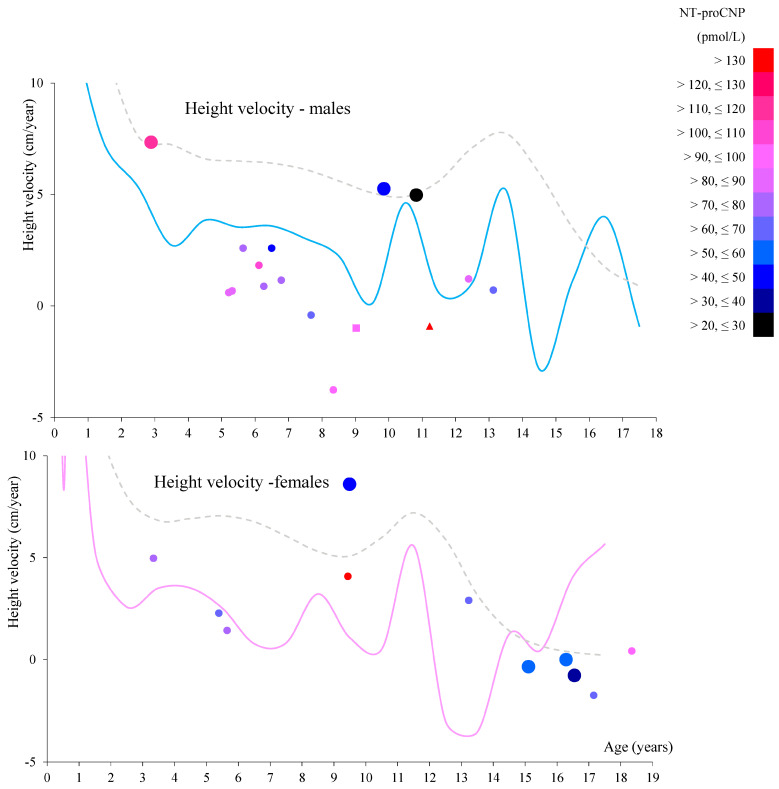
The relationship between each patient’s plasma NT-proCNP level, age, and subsequent height growth rate (height velocity). Height velocity was calculated from two height datasets measured at different times, 7 to 20 months apart. Each dot represents the age of each patient at which the first height was measured (horizontal axis) and the corresponding later height velocity (vertical axis). Round dots represent patients continuously receiving enzyme replacement therapy (ERT) until the second height data were obtained. The square dot represents a patient who had received hematopoietic stem cell transplantation (HSCT) (M08). The triangular dot indicates a patient who never received ERT until his second height data were obtained (M35). Larger round dots represent the attenuated phenotype, while smaller round dots represent the severe phenotype. Each dot is filled with a color corresponding to the plasma NT-proCNP level at the first height measurement. The light blue line shows the 50th percentile for height velocity for male MPS IVA patients, and the pink line shows the 50th percentile for female MPS IVA patients [62]. The gray dotted line shows the 50th percentile for height velocity for healthy males and females.

The relationship between each patient’s plasma NT-proCNP level, age, and subsequent height growth rate (i.e., near-future growth rate) is shown in Figure 3. Higher NT-proCNP levels did not lead to a higher growth rate but to a lower one, especially after the age of 8 years. We could not statistically analyze this trend due to the limited data.

#### 2.3.2. Collagen Type I

We found a statistically significant increase in collagen type I in patients older than 20 years (Table 3, Figure S2A). The correlation between serum and plasma was straightforward, as shown in Figure S3, indicating that the comparison between serum from controls and plasma from patients is feasible and reasonable.

#### 2.3.3. Collagen Type II

We found a significant increase in collagen type II in patients in two age groups, between 5 and 10 years and over 20 years (Table 4, Figure S2B). However, the correlation between serum and plasma was unclear, as shown in Figure S4, which complicated the interpretation of the results for the former age group. In addition, we calculated the partial correlation coefficient between collagen type II and NT-proCNP, controlling for age using the entire age group because collagen type II is a marker of chondrocytes [56,57], and CNP is a growth factor for chondrocytes [36]. However, our data showed no correlation (*r* = 0.042, *p* = 0.69).

#### 2.3.4. Glycosaminoglycans (GAGs)

**Figure 4:**
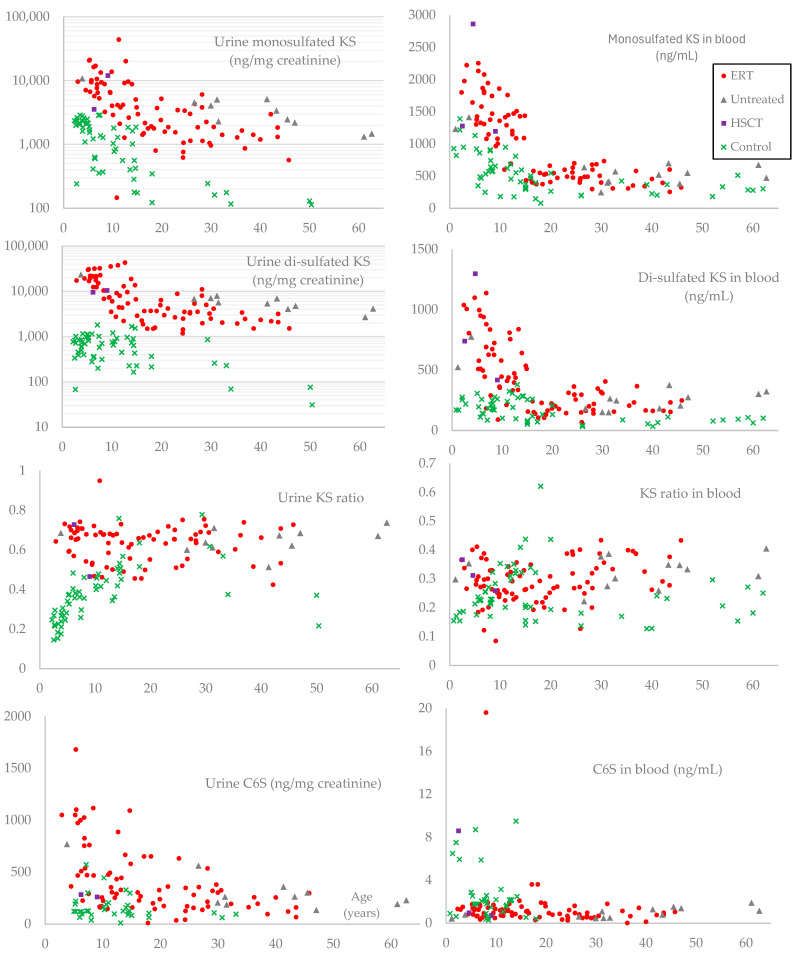
Glycosaminoglycan levels in urine and blood. Mono-sulfated KS, di-sulfated KS, KS ratio (di-sulfated KS to total KS), and C6S levels in urine and blood are summarized in scatter plots. The horizontal axes indicate the age (years) of each patient or control. For urinary mono-sulfated and di-sulfated KS levels, the vertical axes are shown on a base-10 logarithmic scale. Round red dots indicate patients who had ever received ERT. Purple squares indicate patients who received HSCT. Gray triangles represent patients who never received ERT or HSCT. Green x marks represent controls. For blood samples, plasma samples were used for patients and serum for controls.

The results of mono-sulfated KS, di-sulfated KS, KS ratio (di-sulfated KS to total KS), and C6S in urine and blood from patients are summarized in Table 5 and Figure 4. Patients had a higher sulfation level in urinary KS than age-matched controls, except for the group aged between 15 and 20 years. Sulfation levels of urinary KS in normal controls increased with age, while the levels in MPS IVA patients remained constant in all age groups. MPS IVA patients had higher sulfation levels in blood KS than age-matched controls up to 10 years. Sulfation levels of blood KS in normal controls increased until 15 years and then declined with age, whereas levels in MPS IVA patients remained relatively constant across all age groups. Overall, sulfation patterns and age-dependent changes differed between urinary KS and blood KS in both control and MPS IVA patient groups, indicating the difference in the origin of urinary and blood KS.

Age-adjusted Pearson partial correlation coefficients (*r*) or Spearman partial correlation coefficients (*ρ*) between the z-score of height and each significant GAG (mono- and di-sulfated KS and urinary C6S) were calculated and are summarized in Table 6. Both urinary KS showed a negative correlation with the z-score of height with statistical significance.

#### 2.3.5. Effect of ERT on Each Biomarker in Adult Patients Aged 25–50 Years

Two biomarkers, urinary mono-sulfated KS and di-sulfated KS, significantly decreased in patients treated with ERT, whereas other biomarkers showed no significant differences (Table 7).

#### 2.3.6. Transitions in Skeletal Growth and Biomarkers in Pediatric Patients Younger than 12 Years with More than Two Hospital Visits (Table 8)

All patients who met the criteria described in the Materials and Methods Section received ERT (12 patients) or HSCT (2 patients). Height was categorized into percentiles based on the growth chart constructed from the accumulated data of Morquio A patients without ERT or HSCT [7]. Red-colored cells indicate improvement in the percentile category. Blue-colored cells indicate a worsening of the percentile category. Pink-colored cells indicate an increase of more than 6% in the biomarker value. Light blue-colored cells indicate a decrease of more than 7%. Gray-colored cells indicate almost no change in the biomarker over the two points.

Two of the eleven patients who routinely received ERT worsened in the height percentile category. Three showed improvement, and the remaining six were not affected in their height (Table 8). Five out of nine patients with ERT had decreased NT-proCNP levels in plasma, three had increased levels, and one had the same level. Six out of nine patients with ERT had increased blood mono-sulfated KS levels, and three had decreased levels. Nine out of ten patients with ERT had decreased mono-sulfated KS levels in urine, and one had increased level. Six out of nine patients with ERT had increased blood di-sulfated KS levels, and three had decreased levels. Five out of ten patients with ERT had increased urinary di-sulfated KS levels, and five had decreased levels. No correlation was found in two-point changes (decrease, increase, or no change) between NT-proCNP and urinary KS or blood KS.

**Table 8:** NT-proCNP and GAG levels in pediatric patients under 12 years of age.

				Percentile	NT-ProCNP	Mono-Sulfated KS		Di-Sulfated KS		KS Ratio		
		Height	Age	of Height	Plasma	Plasma	Urine	Plasma	Urine	Plasma	Urine	
ID	Sex	(cm)	(Year)		(pmol/L)	(ng/mL)	(ng/mg cre)	(ng/mL)	(ng/mg cre)			Treatment
M52	F		1.84									HSCT
		83	2.16	**25th–50th**	no data	no data	no data	no data	no data	no data	no data	
		No record	2.5		no data	1277.0	no data	739.0	no data	0.3668	no data	
		91.5	3.14	**75th**								
		No record	4.6		64.94	2864.2	no data	1297.2	no data	0.3117	no data	
		104	6.21	**75th–90th**	no data	no data	3564	no data	9502	no data	0.7272	
M51	M	91.5	2.89	**50th–75th**	117.43	1977.9	9658	1005.9	17,357	0.3371	0.6425	ERT
		103.3	4.5	**75th–90th**	90.39	1643.9	7102	1098.5	19,299	0.4006	0.7310	
M16	M	85.6	5.21	3rd–10th	83.29	1305.0	20,783	509.0	30,026	0.2806	0.5910	ERT
		86.2	6.23	3rd–10th	no data	no data	no data	no data	no data	no data	no data	
		No record	6.77	No record	99.67	1818.3	7679	690.5	17,611	0.2752	0.6964	
M15	M	99.5	5.32	50th–75th	85.15	1378.1	21,141	578.0	30,952	0.2955	0.5942	ERT
		100.5	6.81	50th	103.81	2078.6	8783	878.7	21,695	0.2971	0.7118	
M55	F	96.7	5.39	50th–75th	60.13	1426.9	6701	996.6	16,964	0.4112	0.7168	ERT
		99.9	6.8	50th–75th	104.42	1310.0	no data	182.2	no data	0.1221	no data	
M50	F	97	5.65	50th–75th	76.66	2134.8	10,127	949.8	19,754	0.3079	0.6611	ERT
		99.3	7.26	50th–75th	59.80	1079.4	5293	627.5	15,186	0.3676	0.7415	
M21	M	95	6.12	25th–50th	109.48	1328.6	16,376	496.0	21,635	0.2718	0.5692	ERT
		97.6	7.55	25th–50th	90.06	1945.9	13,424	834.9	32,474	0.3003	0.7075	
M46	F	96.6	6.51	50th	no data	no data	no data	no data	no data	no data	no data	ERT
		No record	6.92	No record	no data	no data	6476	no data	12,382	no data	0.6566	
		100.6	8.4	50th	97.35	1470.7	3246	723.3	6846	0.3297	0.6784	
M08	M	96.4	7.86	25th	no data	no data	no data	no data	no data	no data	no data	ERT
		No record	8		no data	1377.9	no data	674.3	no data	0.3286	no data	
			8.07									HSCT
		99	9.03	25th	91.53	1196.0	12,063	418.0	10,503	0.2588	0.4654	
		97.6	10.42	10th–25th	no data	no data	no data	no data	no data	no data	no data	
M22	M	100	8.34	25th–50th	98.16	1746.7	8848	626.6	10,450	0.2640	0.5415	ERT
		94.6	9.78	10th–25th	79.68	1859.7	13,776	579.1	35,514	0.2375	0.7205	
M19	F	98.5	9.44	**25th–50th**	167.19	1081.4	6616	363.0	7330	0.2513	0.5256	ERT
		104.7	10.96	**50th**	168.59	1415.3	4146	411.4	9157	0.2252	0.6883	
M20	F	111.1	9.49	75th–90th	41.26	1005.8	6480	353.7	7416	0.2602	0.5337	ERT
		116	10.06	75th–90th	no data	no data	2903	no data	6026	no data	0.6749	
M31	M	129.5	9.86	**90th**	49.42	1359.5	4043	445.9	3538	0.2470	0.4667	ERT
		137.8	11.44	**90th–97th**	28.52	1760.7	2116	808.5	4436	0.3147	0.6770	

Red-colored cells indicate improvement in the percentile category. Blue-colored cells indicate a worsening of the percentile category. Pink-colored cells indicate an increase of more than 6% in the biomarker value. Light blue-colored cells indicate a decrease of more than 7%. Gray-colored cells indicate almost no change in the biomarker over the two points.

Sulfation levels (KS ratio) in the urine of all patients were higher than in the blood. Nine out of ten patients with ERT had increased sulfation levels in urine, and one had the same level. Four out of nine patients with ERT had increased blood sulfation levels, three had decreased levels, and two had the same level.

#### 2.3.7. Correlation Between NT-ProCNP and Other Biomarkers

The correlation between NT-proCNP and other biomarkers was examined in the three pediatric age groups and is summarized in Table S2. Only plasma and urinary C6S showed correlations with statistical significance (*p* < 0.05) over 13 years of age, suggesting that NT-proCNP can be an independent biomarker from KS.

## 3. Discussion

In this study, we measured several biomarkers in 60 MPS IVA patients enrolled in the Morquio A natural history program (clinicaltrial.gov: NCT05284006) to evaluate the significance of each biomarker concerning growth failure. Fifty-one patients (85%) received ERT, and two (3.3%) received HSCT. Two (3.3%) pediatric patients had not yet started ERT because they were newly diagnosed. Six (10%) adult patients had never received ERT despite its approval in the United States. Judging from their heights, ERT did not show apparent effects for skeletal dysplasia, including bone growth (Figure 1, Table 8), as indicated by the previous reports [62,63,64,65,66,67]. However, it should be noted that we could not statistically compare with and without ERT in pediatric patients due to the limited number of pediatric patients without ERT, which is a limitation of our research. We identified four novel mutations through genetic testing: one was a deletion resulting in a frameshift, another was a mutation at a splice donor site, and the remaining two were missense mutations predicted to be disease-causing by two bioinformatics tools. In 10 patients, we found only one mutation on one allele. We sequenced only the exons and exon-intron boundaries of the *GALNS* gene and did not search for large deletions, thereby limiting our genetic testing.

CNP is significantly involved in both vascular homeostasis and bone growth, which is mainly controlled by CNP secreted from vascular endothelial cells [68] and chondrocytes in growth plates [35], respectively. It plays a key role in endochondral ossification, crucial for forming and growing long bones [29]. CNP primarily affects bone growth by signaling through the chondrocyte receptor NPR-B, encoded by the *NPR2* gene, which induces cell division and differentiation [38].

Recent research indicated that CNP and NT-proCNP levels are significantly elevated in several skeletal dysplasias (achondroplasia, hypochondroplasia, thanatophoric dysplasia, and Maroteaux type of acromesomelic dysplasia) caused by mutations in the *FGFR3* (fibroblast growth factor receptor 3) or *NPR2* (natriuretic peptide receptor 2) gene [69]. These genes are closely related to each other and to extracellular CNP, playing a crucial role in cell proliferation and differentiation. Substantial progress has been made in recent years in the study of CNP and their role in skeletal disorders, particularly achondroplasia, which is caused by a gain-of-function mutation in *FGFR3* that results in constitutive activation of FGFR3 and the downstream MEK/ERK MAPK pathway [70,71]. The MEK/ERK MAPK pathway is a signaling pathway that regulates various cellular processes, including cell growth, proliferation, and differentiation [72,73,74]. Elevated levels of CNP and NT-proCNP in achondroplasia are thought to be a natural response of the human body to this overactivation of the MEK/ERK MAPK pathway in chondrocytes, which functionally inhibits the effect of CNP on chondrocytes via NPR-B encoded by *NPR2* [69,75]. In this paper, we have identified the elevation of NT-proCNP in MPS IVA patients. The gene responsible for MPS IVA is *GALNS*, which is not closely related to *FGFR3* or *NPR2.* Therefore, this is the first report to identify the elevation of NT-proCNP in patients with a disease unrelated to mutations in the *FGFR3* or *NPR2* genes.

Recent research has not only identified NT-proCNP as a potential biomarker but also highlighted the therapeutic potential of CNP. The development of drugs such as Vosoritide, a CNP analog approved to induce bone growth in achondroplasia [70,71,76], underscores the potential of CNP as a therapeutic agent against the growth impairment of MPS. This potential has already been demonstrated in MPS IVA and VII mouse models [77,78]. Furthermore, a Phase I/II clinical trial of the CNP analog in MPS IVA and VI patients has been initiated to explore further these therapeutic possibilities (ClinicalTrials.gov ID: NCT05845749) and inform the scientific community about the latest developments in MPS research.

NT-proCNP is a peptide fragment derived from the precursor protein of CNP. The *NPPC* (natriuretic peptide precursor C) gene expresses a precursor protein known as preproCNP. PreproCNP undergoes proteolytic cleavage to remove the signal peptide and to form proCNP [42]. Furin, one of the proprotein convertases, cleaves NT-proCNP from proCNP in the trans-Golgi network to produce the active CNP [40,41,79,80,81]. After the cleavage of proCNP, active CNP is secreted into the extracellular space via exocytosis [42]. For NT-proCNP, we found no clear mechanism of how it is secreted by cells; however, these processes should occur primarily in neurons, glial cells [30,31,32], vascular endothelial cells [33], and chondrocytes in cartilage [34,35]. The half-life of CNP in the blood is short, about 2–3 min, as NPRs take up CNP, and such a short half-life helps to adapt to frequent changes in circulatory status [41,43,82]. In the case of NT-proCNP, there is no clear pathway to remove it. As a result, the normal concentration ranges of these two molecules in healthy children are significantly different: CNP is 0.5~3 pmol/L, and NT-proCNP is 15~60 pmol/L (74.8~299 pg/mL) [45]. This difference makes NT-proCNP easier to detect and quantify than CNP. Considering that bone growth is a slower and more gradual phenomenon than the adaptation of the circulatory system, NT-proCNP is expected to be a better biomarker than CNP for correlating human skeletal symptoms in MPS. This leads us to select NT-proCNP as a potential surrogate biomarker for future studies.

Our data demonstrated that NT-proCNP was significantly elevated in the plasma of MPS IVA patients in all comparable age groups. In addition, the concentrations in patients negatively correlated with height z-score, the most reliable indicator of skeletal growth failure [83,84]. The source of this elevated NT-proCNP (presumably, CNP as well) remains unknown. The primary sources of these peptides are neurons, glial cells [30,31,32], vascular endothelial cells [33], and chondrocytes in cartilage [34,35], but the exact percentage of production at each site under normal conditions remains uncertain. The CNP production by growth plate chondrocytes is the most critical factor for bone growth, as evidenced by the growth impairment observed in mice where the *Nppc* gene has been selectively knocked out in chondrocytes [35]. Even when plasma CNP levels were significantly reduced by knocking out the *Nppc* gene selectively in mouse vascular endothelial cells, Moyes et al. did not observe any growth failure [68].

Based on previous and current results, we propose potential hypotheses (factors) contributing to plasma NT-proCNP elevation in MPS IVA patients.

1. The expression of the *NPPC* gene is upregulated in MPS IVA patients to compensate for growth impairment or tissue resistance to CNP, as seen in achondroplasia, hypochondroplasia, and thanatophoric dysplasia [69]. A feedback mechanism in patients with growth failure contributes to the higher expression of CNP. This feedback mechanism could involve vascular endothelial cells as a source of excessive CNP and NT-proCNP secretion.
2. CNP and NT-proCNP are leaking from damaged chondrocytes in MPS IVA patients. The secretion or leakage of NT-proCNP from damaged chondrocytes in the growth plates can enter the bloodstream, increasing its concentration in the blood.
3. NT-proCNP clearance is delayed due to a systemic disorder of MPS IVA.

A combination of multiple factors may account for the elevation of NT-proCNP. With age, the contribution factor could vary. Further research is needed to investigate the regulation of CNP expression (at both mRNA and protein levels) and clarify this paradoxical phenomenon.

Focusing on patients who had two data points at different ages prior to peak pubertal growth spurt (all of whom had received ERT during these two periods), NT-proCNP levels increased in 5 of 10 boys and 2 of 3 girls (Figure 2) while urinary mono-sulfated KS was reduced in 9 out of 10 patients, as shown in Table 8. This finding indicates that plasma NT-proCNP is not a pharmacodynamic biomarker like urinary KS prior to the onset of pubertal growth spurt. Therefore, we hypothesize that reduced NT-proCNP can be more directly linked to improving bone growth than reducing total urinary KS. Further studies will confirm whether NT-proCNP is an excellent surrogate biomarker that correlates with clinical improvement in the skeletal system.

Collagen type I is the most abundant collagen in the human body. It is a fibrous protein that forms a significant part of the extracellular matrix in various tissues, including skin, tendons, ligaments, and, importantly, bone [47,48,49]. It provides a scaffold for bone mineralization and forms a fibrous network that serves as the structural framework for bone tissue [50,51,52]. This network supports the deposition of hydroxyapatite crystals, primarily composed of calcium and phosphate [85,86]. The interaction between collagen fibers and these crystals gives bones strength and rigidity. Procollagen Type I N-Terminal Propeptide (P1NP) and C-Terminal Telopeptide of Type I Collagen (CTX-I) are two major peptides well studied in several diseases related to collagen type I metabolism. P1NP reflects collagen synthesis [87], while CTX-I reflects collagen degradation [88]. Elevated levels of these peptides in human blood or urine are associated with diseases or conditions related to increased bone turnover (such as Paget’s disease [89,90], osteoporosis [91], or hyperparathyroidism [92,93]), fibrotic processes (such as liver fibrosis and cirrhosis [94,95]), or cancer (bone metastases [96,97] or multiple myeloma [98]).

Several papers reported that the expression level of mRNA encoding collagen type I increased in chondrocytes from human MPS IVA patients [53,54]. Comprehensive proteomic analysis of 6-week-old mouse femurs also showed that collagen alpha-1 and 2 (Uniprot IDs: P11087 and Q01149) increased in MPS IVA [99]. Therefore, we predicted that patients would present higher concentration levels than the control group. However, only adult patients showed such differences. Other age groups showed lower concentrations in patients. Since human collagen type I has a molecular weight of approximately 300 kDa, much larger than NT-proCNP, surrounded by hydroxyapatite in bone [85,86,100], it could be difficult for collagen type I to escape from bone and cartilage and enter the bloodstream compared to NT-proCNP, thereby preventing higher concentrations in the blood of patients. We should have measured smaller peptides related to collagen type I, as in other diseases described above.

Collagen type II is a specific type of collagen found predominantly in cartilage [55] and is a marker for chondrocytes [56,57]. It is also present in the vitreous humor of the eye and in the nucleus pulposus of intervertebral discs [101]. Like other collagens, type II collagen is a fibrous protein that forms a triple-helical structure, providing tensile strength and structural integrity to tissues [59]. Its primary role in forming and maintaining cartilage makes it crucial for joint health, enabling smooth and pain-free movement [102,103]. Additionally, its presence on the intervertebral discs and the vitreous body of the eye underscores its importance in maintaining flexibility and structural support in these tissues. Blood procollagen II C-terminal propeptide (CPII) and/or urinary C-terminal telopeptide of type II collagen (CTX-II) are currently the two main biomarkers of type II collagen metabolism in osteoarthritis [104,105,106], joint injuries [107], and Kashin–Beck disease (KBD) [108]. CPII reflects the synthesis of type II collagen during cartilage repair and remodeling [109,110,111], while CTX-II is a marker of type II collagen breakdown, reflecting cartilage catabolism [106,112].

De Franceschi et al. and Dvorak-Ewell et al. reported decreased expression of mRNA encoding collagen type II in chondrocytes from human MPS IVA patients, in contrast to collagen type I [53,54]. The same phenomenon was also observed in mouse models of MPS type I and type VII [113,114]. These findings suggest that the decrease in collagen type II may be a potential biomarker for MPS types I, IVA, and VII. In contrast, proteomic analysis of 6-week-old mice revealed a statistically significant increase in collagen alpha-1(II) chain (Uniprot ID: P28481) in the MPS IVA model [99]. This discrepancy in collagen type II expression levels between humans and mice warrants further investigation. Our results showed a statistically significant increase in patients aged 5 to 10 years and over 20 years. This seems to support the results of the latter proteomic analysis [99]. However, due to the poor correlation between the measured concentration in serum and plasma, as shown in Figure S4, it is too early to draw conclusions for patients aged 5 to 10 years.

We also calculated the correlation coefficient between collagen type II and NT-proCNP, controlling for age, to evaluate the effect of excessive CNP expression on chondrocytes in patients. NT-proCNP concentration would indicate the total expression level of CNP in a subject’s body. Collagen type II is a marker of chondrocytes [56,57]. Therefore, elevated NT-proCNP levels could correlate positively with collagen type II levels. However, we found no correlation (*r* = 0.042, *p* = 0.69), suggesting that the increased CNP secretion in patients is insufficient to compensate for the damage to chondrocytes in growth plates.

GAG levels are well-established biomarkers for MPS, and the difference between plasma and serum is negligible, making our comparison between controls and patients reasonable [115]. Our results were consistent with the previous reports [22,116]. However, to our knowledge, we could not find any prior report that quantitatively correlates GAG levels with the severity of skeletal symptoms. Therefore, we correlated each GAG with the z-score of height, one of the quantitative indicators of growth failure. Since each GAG level except for plasma C6S tended to decrease with age up to 20 years (Figure 4), we calculated partial correlation coefficients controlling for age, then found a moderate negative correlation in urinary mono- and di-sulfated KS (Table 6). These two biomarkers also showed statistical differences between adult patients with and without ERT (Table 7), making urinary KS a more important prognostic biomarker than C6S in MPS IVA.

We established the KS assay by LC-MS/MS, measuring both mono- and di-sulfated KS in age-matched controls and MPS IVA patients [13,14,18,115]. Our previous studies identified differences in age and sulfation between urine and blood KS in MPS IVA and healthy controls, suggesting the importance of measuring both types of KS in MPS IVA [8,14]. Urinary KS is considered a pharmacodynamic biomarker that does not accurately reflect the therapeutic effect on skeletal symptoms in patients receiving ERT [26]. Hendriksz et al. presumably measured both KSs (mono-sulfated and di-sulfated) and calculated the total KS [117]. In this study, we observed the transition of these two types of KS during ERT or after HSCT in the most critical age group for treatment of skeletal growth, namely, those under 10 years old. Interestingly, a decrease in urinary di-sulfated KS was observed in 5 out of 10 patients, whereas 9 out of 10 patients showed a decrease in mono-sulfated KS (Table 8). Based on this result, we assume that urinary di-sulfated KS may not be a pharmacodynamic biomarker like mono-sulfated KS. Furthermore, blood di-sulfated KS showed more pronounced differences from controls in most age groups (Figure 4). Further investigations are needed to clarify the significance of di-sulfated KS.

## 4. Materials and Methods

Subjects: MPS IVA patients receiving care at Nemours Children’s Health in Delaware and participating in the natural history program (Non-invasive Functional Assessment and Pathogenesis of Morquio A, clinicaltrial.gov: NCT05284006, NIH funding number: 1R01HD102545-01A1) were enrolled. The diagnosis of MPS IVA was confirmed by deficient enzyme activity of <5% of normal activity measured in plasma, leukocytes, or fibroblasts before enrollment in this clinical trial. Before any study procedures, written informed consent was obtained from all participants. A total of 60 participants formalized their consent for the research study. The z-score of each patient’s height during sample collection was calculated based on the CDC Growth Chart [84]. It was also categorized into percentiles based on the growth chart constructed from the accumulated data of Morquio A patients without ERT or HSCT (hematopoietic stem cell transplantation) [7]. If a patient had two or more height measurements taken at different times, the height growth rate (annual height velocity) was calculated using two data points.

Genotyping: Genomic DNA was extracted from the white blood cells and/or skin fibroblasts. The 14 exons of the *GALNS* gene were amplified by polymerase chain reaction (PCR) and sequenced using the Sanger method to identify pathogenic mutations. PCR conditions and primer pairs used are summarized in Table S3. Mutations were annotated based on the reference sequence NM_000512.5.

Control Specimens: Serum or urine samples from individuals without skeletal dysplasia at Shimane University Hospital (Japan) were provided as control samples for this research. The age ranged from 0 days to 18 years for serum and from 5 to 33 years for urine. Additionally, blood samples were collected from 7 adults without skeletal dysplasia working in our laboratory, and both plasma and serum were tested to confirm the correlation between serum and plasma for collagen type I and II levels. Written informed consent was obtained from all individuals (IRB# 750932).

ELISA-based method for the measurement of collagen type I, II, and NT-proCNP: The Human Collagen Type I (COL1) ELISA Kit (Cat# EKU03297-96T, BIOMATIK, Kitchener, Canada), the Human Collagen Type II, Col II ELISA Kit (Cat# EKC40379, BIOMATIK), and the NT-proCNP ELISA kit (Cat# BI-20812, BIOMEDICA, Vienna, Austria) were utilized to measure the concentration of each biomarker in patient plasma and serum, control serum from children, and control plasma and serum from adults according to the manufacturer’s instructions. Each kit used a quantitative sandwich enzyme immunoassay technique. The absorbance of each well was measured using FLUOstar Omega (BMG LABTECH, Ortenberg, Germany).

Glycosaminoglycans (GAGs) Analysis by LC-MS/MS: Mono-sulfated KS, di-sulfated KS, and C6S in the plasma and urine samples from patients were measured according to our established method [22,118]. Briefly, 10 μL of each serum, plasma, urine sample, or standard was mixed with 90 μL of 50 mM Tris-hydrochloric acid buffer (pH 7.0) in wells of AcroPrep™Advance 96 Well Filter Plates equipped with ultrafiltration Omega10K membrane filters (PALL Corporation, Port Washington, NY, USA). A cocktail of 30 μL of recombinant chondroitinase ABC and keratanase II (each enzyme at 1 mU/sample) and internal standard solution (5 μg/mL) was added to each well, followed by the addition of 70 μL of 50 mM Tris-hydrochloric acid buffer. After overnight incubation at 37 °C, the filter plate was centrifuged for 20 min at 2500× *g*. The filtered samples were injected into our 1290 Infinity liquid chromatography system with a 6460 triple quad mass spectrometer (Agilent Technologies, Palo Alto, CA, USA). Levels of GAGs in urine samples were normalized to creatinine, which was measured with a Creatinine (urinary) Colorimetric Assay Kit (Cayman Chemical, Ann Arbor, MI, USA). The serum and urine of control subjects were also measured in the same way.

Effect of ERT on Each Biomarker: We compared the difference in each biomarker between patients treated with ERT and patients who had not been treated with ERT. Since most patients in the latter group were between 25 and 50 years old, we used only this age range.

Transitions in skeletal growth and biomarkers in pediatric patients younger than 12 years with more than two hospital visits: To explore a correlation between significant biomarkers, skeletal development, and treatment (ERT or HSCT) in growing pediatric patients, we summarized the data from patients who provided their first specimen before the age of 10 years in Table 8.

Statistical Analysis: For urinary mono- and di-sulfated KS, the Mann–Whitney U test was used to determine statistical differences between subjects and controls. For other biomarkers, Welch’s *t*-test was performed. Both tests were calculated with GraphPad Prism version 10.2.3. R-4.4.3 for Windows was used to calculate correlation coefficients or partial correlation coefficients, controlling for age, to reveal the relationship between each factor [119,120]. To evaluate correlations involving urinary mono- or di-sulfated KS, the Spearman correlation coefficient was calculated. For correlations not involving these two biomarkers, the Pearson correlation coefficient was used.

## 5. Conclusions

In MPS IVA patients, an elevated blood NT-proCNP level is a promising biomarker closely associated with growth failure. Collagens I and II are less promising than other biomarkers, such as urinary and blood KS. In addition, KS should be evaluated in more detail in MPS, as di-sulfated KS may not be a simple pharmacodynamic biomarker in patients receiving ERT.

## Figures and Tables

**Table 1:** Characteristics of all participants.

Patient			Height	Age	Genotype		
ID	Sex	Race	(Z-Score)	(Year)	Allele 1	Allele 2	Treatment
M01	F	White or Caucasian	−6.34	17.16	c.740G>A (G247D)	c.901G>T (G301C)	ERT
			−6.73	18.70			
M02	F	Chinese		38.57	c.953T>G (M318R)	c.1567T>G (X523EextX93)	ERT
				40.00			
M03	F	White or Caucasian		24.62	c.448delC (H150Tfs*3)	c.651_652insG (K218Efs*45)	ERT
M04	F	White or Caucasian		42.12	c.675dupC (F226Lfs*37)	unknown	ERT
				43.51			
M05	F	White or Caucasian	−9.93	18.35	c.346G>A (G116S)	c.1156C>T (R386C)	ERT
			−9.76	19.78			
M06	F	White or Caucasian	−8.52	19.89	c.346G>A (G116S)	c.1156C>T (R386C)	ERT
				21.29			
M07	M	White or Caucasian		32.25	c.337A>T (I113F)	c.1171A>G (M391V)	No ERT for 2 years and 8 months
M08	M	White or Caucasian	No record	8.00	c.122T>A (M41K)	c.122T>A (M41K)	ERT
			−6.09	9.03			Received HSCT 1 year ago
M10	M	White or Caucasian	1.22	10.82	c.338T>C (I113T)	c.1219A>C (N407H)	ERT
			1.01	12.37			
M11	F	White or Caucasian	−5.36	15.11	c.421T>A (W141R)	unknown	ERT
			−5.53	16.55			
M12	F	White or Caucasian		26.65	c.697G>A (D233N)	c.1034T>C (L345P)	No ERT for 4 years and 4 months
				28.15			
M13	M	Black or African American	−7.52	13.13	c.251C>A (A84E)	c.319G>A (A107T)	ERT
			−6.75	14.84			
M14	F	White or Caucasian	−5.7	16.29	c.740G>A (G247D)	unknown	ERT
			−5.7	17.83			
M15	M	White or Caucasian	−2.39	5.32	c.498delC (F167Lfs*32)	c.901G>T (G301C)	ERT
			−3.84	6.81			
M16	M	Filipino	−5.09	5.21	c.228C>A (N76K)	c.1480A>G (p.M494V)	ERT
			No record	6.77			
M19	F	Black or African American	−6.49	9.44	c.245C>T (S82L)	unknown	ERT
			−5.64	10.96			
M20	F	White or Caucasian	−4.09	9.49	c.498delC (F167Lfs*32)	c.1474G>A (A492T)	ERT
			−3.53	10.06			
M21	M	White or Caucasian	−4.08	6.12	c.651_652insG (K218Efs*45)	c.1159G>A (G387S)	ERT
			−5.22	7.55			
M22	M	White or Caucasian	−5.45	8.34	c.651_652insG (K218Efs*45)	c.1159G>A (G387S)	ERT
			−7.24	9.78			
M25	M	White or Caucasian		41.34	c.155C>T (P52L)	c.337A>T (I113F)	Never received ERT
				43.28			
M26	M	Filipino	−7.03	12.39	c.93delC (N32Tfs*97)	c.946G>A (G316R)	ERT
			No record	13.89			
M28	F	Mixed		31.20	c.868G>A (G290S)	c.868G>A (G290S)	Never received ERT
		Japanese and Caucasian		32.75			
M30	F	White or Caucasian		43.52	c.121-12T>C	c.121-12T>C	ERT
M31	M	Black or African American	−1.29	9.86	c.935C>G (T312S)	c.1520G>T (C507F)	ERT
			−1.11	11.44			
M32	F	White or Caucasian		45.50	c.121-12T>C	c.121-12T>C	Never received ERT
				47.03			
M33	F	White or Caucasian		29.94	c.1012C>T (Q338X)	c.1171A>G (M391V)	Never received ERT
				31.48			
M34	M	White or Caucasian		28.17	c.121A>T (M41L)	c.121A>T (M41L)	ERT
				29.69			
M35	M	White or Caucasian	−6.86	11.23	c.139G>A (G47R)	c.1156C>T (R386C)	No ERT for 4 years
			−7.69	12.64			
M36	F	Asian Indian		25.72	c.346G>A (G116S)	c.346G>A (G116S)	ERT
				27.27			
M37	M	Asian Indian	−10.12	19.01	c.346G>A (G116S)	c.346G>A (G116S)	ERT
				20.56			
M38	F	White or Caucasian	−5.63	16.04	c.1559G>A (W520X)	unknown	ERT
M39	F	White or Caucasian	−3.54	12.08	c.1559G>A (W520X)	unknown	ERT
M41	M	White or Caucasian		35.29	c.121A>T (M41L)	c.498delC (F167Lfs*32)	ERT
				36.86			
M42	F	White or Caucasian		28.24	c.860C>T (S287L)	c.1055T>C (L358P)	ERT
				29.95			
M43	M	White or Caucasian		29.04	c.121A>T (M41L)	c.901G>T (G301C)	ERT
				30.52			
M44	F	White or Caucasian		24.28	c.1171A>G (p.M391V)	c.502G>A (p.G168R)	ERT
				25.85			
M45	F	White or Caucasian		61.08	c.740G>A (G247D)	c.761A>G (Y254C)	Never received ERT
				62.66			
M46	F	White or Caucasian	No record	6.92	c.167C>A (T56N)	c.502G>A (G168R)	ERT
			−5.54	8.40			
M47	F	Some other race		24.33	c.139G>A (G47R)	c.466T>C (F156L)	ERT
				25.78			
M48	F	White or Caucasian	−7.11	13.23	c.139G>A (G47R)	c.466T>C (F156L)	ERT
			−7.66	14.68			
M49	M	White or Caucasian	−6.44	7.68	c.1156C>T (R386C)	c.1156C>T (R386C)	ERT
M50	F	White or Caucasian	−3.33	5.65	c.451C>A (P151T)	c.477G>A (W159X)	ERT
			−4.81	7.26			
M51	M	White or Caucasian	−0.9	2.89	c.451C>A (P151T)	c.477G>A (W159X)	ERT
			−0.52	4.50			
M52	F	Multiple Races	No record	2.50	c.651_652insG (K218Efs*45)	unknown	Received HSCT 11 months ago
			No record	4.60			
			−2.54	6.21			
M53	F	Chinese		23.20	c.1482+5G>C	c.1498G>T (G500C)	No ERT for 13 months
				24.37			ERT
M54	F	White or Caucasian		36.26	not tested	not tested	No ERT for 5 years and 9 months
M55	F	White or Caucasian	−3.04	5.39	c.1339G>C (D447H)	unknown	ERT
			−4.2	6.80			
M56	F	White or Caucasian	−1.44	3.79	c.740G>A (G247D)	c.1451C>A (P484H)	Never received ERT
M57	M	White or Caucasian	0.41	1.13	c.740G>A (G247D)	c.1451C>A (P484H)	Never received ERT
M58	M	White or Caucasian	−4.38	6.78	c.1012C>T (Q338X)	c.181C>G (R61G)	ERT
M59	M	White or Caucasian	−4.14	6.26	c.337A>T (p.I113F)	c.901G>T (p.G301C)	ERT
M60	F	Some other race		26.58	c.901G>T (G301C)	c.1156C>T (R386C)	Never received ERT
M63	F	White or Caucasian		22.72	c.331C>T (Q111X)	c.1365-2A>G	ERT
M64	M	Black or African American	−3.74	5.64	c.633+1G>C	c.1558T>C (W520R)	ERT
M65	M	Black or African American	−2.9	6.50	c.633+1G>C	c.1558T>C (W520R)	ERT
M66	F	White or Caucasian	−1.56	3.34	c.1156C>T (R386C)	unknown	ERT
M67	M	White or Caucasian	−1.55	11.58	c.1171A>G (M391V)	c.331C>T (Q111X)	ERT
M68	M	White or Caucasian		45.78	c.1156C>T (R386C)	c.181C>T (R61W)	ERT
M69	M	Asian	−3.03	2.33	c.106_111del (p.L36_L37del)	c.1201C>T (H401Y)	ERT
M71	M	White or Caucasian	−3	9.12	c.1219A>C (N407H)	unknown	ERT

**Table 2:** The partial correlation coefficient between NT-proCNP and the z-score of height, controlling for age, across three age groups.

Age Group	Number of Subjects	Partial Correlation Coefficient	*p*-Value
≤8 y	20	0.190	0.4355
>8, ≤13 y	17	−0.568	0.0217
>13, ≤20 y	15	−0.837	1.865 × 10^−4^

**Table 3:** Comparison of collagen type I levels in five age groups.

Age	No.	Mean	SD	Maximum	Minimum	Mean Age	*p*-Value	Specimen
Control								
≤1 y	1	2803.4	0	2803.4	2803.4	0.25		Serum
>1, ≤5 y	4	2715.5	2400.5	6397.5	329.6	2.51		Serum
>5, ≤10 y	6	1019.0	481.2	2039.0	566.0	7.19		Serum
>10, ≤15 y	5	1572.0	2123.6	5734.8	32.9	11.98		Serum
>15, ≤20 y	1	996.1	0	996.1	996.1	15.99		Serum
>20 y	7	76.6	18.6	106.3	49.3	39.57		Plasma
MPS IVA								
>1, ≤5 y	8	1022.4	681.8	2654.1	490.7	3.05	0.31	Plasma
>5, ≤10 y	24	679.4	469.3	1630.2	50.3	7.38	0.19	Plasma
>10, ≤15 y	14	752.4	398.1	1722.0	381.2	12.52	0.48	Plasma
>15, ≤20 y	11	323.9	314.8	1161.6	67.0	17.70	N/A	Plasma
>20 y	41	125.7	92.1	383.0	24.5	33.35	0.0046	Plasma

**Table 4:** Comparison of collagen type II levels in five age groups.

Age	No.	Mean	SD	Maximum	Minimum	Mean Age	*p*-Value	Specimen
Control								
>1, ≤5 y	18	129.03	92.41	324.99	11.70	2.68		Serum
>5, ≤10 y	12	113.70	53.62	231.40	36.66	6.97		Serum
>10, ≤15 y	7	85.64	78.26	253.57	16.66	12.21		Serum
>15, ≤20 y	2	43.85	30.83	74.68	13.02	16.50		Serum
>20 y	7	31.90	23.13	76.23	8.98	39.57		Plasma
MPS IVA								
>1, ≤5 y	7	105.80	74.15	240.58	29.44	3.23	0.55	Plasma
>5, ≤10 y	21	234.54	233.64	885.55	15.93	7.30	0.037	Plasma
>10, ≤15 y	14	70.07	59.39	208.32	17.03	12.52	0.67	Plasma
>15, ≤20 y	11	131.71	181.03	534.18	12.77	17.70	0.21	Plasma
>20 y	40	87.15	103.81	438.58	6.10	33.54	0.0097	Plasma

**Table 5:** Summary of GAG levels in five age groups.

Age Group	Number of Samples	Mean Age	Mono-Sulfated KS	Di-Sulfated KS	KS Ratio (%)	C6S	Number of Samples	Mean Age
Control	(urine)							
>1, ≤5 y	18	3.47	2128 ± 618	663 ± 263	23.4 ± 4.6	N/A	0	N/A
>5, ≤10 y	14	6.48	1380 ± 833	768 ± 439	36.8 ± 5.6	164.7 ± 129.7	14	6.48
>10, ≤15 y	15	12.75	971 ± 574	802 ± 413	46.5 ± 9.4	178.1 ± 104.1	15	12.75
>15, ≤20 y	3	17	215 ± 96	271 ± 69	57.2 ± 4.9	83.3 ± 17.2	3	17
>20 y	6	37.92	157 ± 45	255 ± 283	48.7 ± 18.6	88.0 ± 19.8	3	31.03
MPS IVA	(urine)							
>1, ≤5 y	3	3.73	9202 ± 1563 **	20,081 ± 2603 **	68.6 ± 3.6 ***	727 ± 282		
>5, ≤10 y	23	7.24	9904 ± 5024 ***	18,225 ± 9116 ***	63.9 ± 8.3 ***	633 ± 398 ***		
>10, ≤15 y	15	12.36	8143 ± 10,725 ***	12,343 ± 11,876 ***	65.1 ± 11.1 ***	465 ± 247 ***		
>15, ≤20 y	11	17.7	2340 ± 1178 **	3091 ± 1509 **	56.7 ± 8.0	289 ± 189 **		
>20 y	39	33.56	2267 ± 1391 ***	4033 ± 2286 ***	64.4 ± 7.7	245 ± 131 ***		
Control	(serum)							
>1, ≤5 y	4	2	1095 ± 223	229 ± 40	17.4 ± 1.3	5.15 ± 2.67	4	2
>5, ≤10 y	17	7.18	660 ± 220	197 ± 59	23.3 ± 3.6	2.34 ± 2.01	17	7.18
>10, ≤15 y	10	12.35	545 ± 235	248 ± 88	32.3 ± 5.0	2.81 ± 2.29	10	12.35
> 15, ≤20 y	10	15.9	322 ± 123	118 ± 73	27.8 ± 14.7	0.49 ± 0.06	2	17
>20 y	15	42.2	314 ± 109	86 ± 43	21.4 ± 7.8	N/A	0	N/A
MPS IVA	(plasma)							
>1, ≤5 y	8	3.13	1805 ± 514 *	910 ± 229 ***	33.7 ± 4.1 ***	1.97 ± 2.53	8	3.13
>5, ≤10 y	24	7.38	1522 ± 369 ***	597 ± 258 ***	27.2 ± 7.7 *	1.97 ± 3.88	22	7.33
>10, ≤15 y	14	12.52	1257 ± 306 ***	509 ± 181 ***	28.4 ± 4.3	1.12 ± 0.41	14	12.52
>15, ≤20 y	11	17.7	482 ± 90 **	166 ± 45	25.5 ± 4.6	1.65 ± 1.04	11	17.7
>20 y	41	33.35	493 ± 131 ***	234 ± 79 ***	32.1 ± 6.9 ***	0.85 ± 0.43	40	33.54

For C6S, the number of available control samples was less than for KS, so the “number of samples” and “mean age” differed from those of KS. GAGs that showed a significant increase in patients are marked with an asterisk (* *p* < 0.05, ** *p* < 0.01, *** *p* < 0.001). No GAGs showed a significant decrease in MPS IVA patients.

**Table 6:** Pearson or Spearman partial correlation coefficient (*r* or *ρ*) between each GAG and the z-score of height of patients under 20 years, controlling for age.

	*n*	*r* or *ρ*	*p*
Urine di-sulfated KS	49	*ρ* = −0.637	1.13 × 10^−6^
Urine mono-sulfated KS	49	*ρ* = −0.645	7.53 × 10^−7^
Plasma di-sulfated KS	52	*r* = −0.048	0.740
Plasma mono-sulfated KS	52	*r* = −0.186	0.192
Urine C6S	49	*r* = −0.282	0.0517

**Table 7:** Differences in each biomarker between adult patients aged 25–50 years with and without ERT.

	Biomarker	Group	Mean Age	Number of Subjects	Mean	SD	*p*-Value
Urine	C6S	No ERT	37.04	8	285	123	0.38
		ERT	33.49	21	237	109	
	Mono-sulfated KS	No ERT	37.04	8	3648	1146	0.0023
		ERT	33.49	21	2005	1320	
	Di-sulfated KS	No ERT	37.04	8	6102	1247	0.0012
		ERT	33.49	21	3540	2254	
	KS ratio	No ERT	37.04	8	0.631	0.057	0.74
		ERT	33.49	21	0.641	0.088	
Plasma	C6S	No ERT	36.57	9	0.913	0.400	0.57
		ERT	33.49	21	0.814	0.428	
	Mono-sulfated KS	No ERT	36.57	9	492	134	0.58
		ERT	33.15	22	460	135	
	Di-sulfated KS	No ERT	36.57	9	226	68.4	0.89
		ERT	33.15	22	221	84.0	
	KS ratio	No ERT	36.57	9	0.317	0.053	0.79
		ERT	33.15	22	0.324	0.076	
	NT-proCNP	No ERT	36.57	9	32.5	6.54	0.77
		ERT	33.15	22	31.6	8.63	
	Collagen type I	No ERT	36.57	9	165.4	118.8	0.32
		ERT	33.15	22	117.2	90.8	
	Collagen type II	No ERT	36.57	9	98.7	84.3	0.63
		ERT	33.49	21	79.5	116.6	

## Data Availability

All available data are included in this article.

## Supplementary Materials

The following supporting information can be downloaded at: https://www.mdpi.com/article/10.3390/ijms26104940/s1.

## Abbreviations

The following abbreviations are used in this manuscript:

MPS	mucopolysaccharidosis
GAG	glycosaminoglycan
CNP	C-type natriuretic peptide
NT-proCNP	N-terminal pro-C-type natriuretic peptide
KS	keratan sulfate
C6S	chondroitin-6-sulfate
GALNS	N-acetylgalactosamine-6-sulfate sulfatase
ERT	enzyme replacement therapy
NPR-B	natriuretic peptide receptor B
PKG	protein kinase G
HSCT	hematopoietic stem cell transplantation
FGFR3	fibroblast growth factor receptor 3
NPR2	natriuretic peptide receptor 2
NPPC	natriuretic peptide precursor C
P1NP	Procollagen Type I N-Terminal Propeptide
CTX-I	C-Terminal Telopeptide of Type I Collagen
CPII	procollagen II C-terminal propeptide
CTX-II	C-terminal telopeptide of type II collagen
KBD	Kashin–Beck disease
PCR	polymerase chain reaction
NIH	National Institutes of Health
