# Supplementary material for: Identification of Surrogate Biomarkers for Mucopolysaccharidosis Type IVA
Source: Int J Mol Sci. 2025 May 21;26(10):4940. doi: 10.3390/ijms26104940 (PMC12112068; doi:10.3390/ijms26104940)
Supplement: Supplementary file 1 [file ijms-26-04940-s001.zip › ijms-3624959-supplementary.pdf]

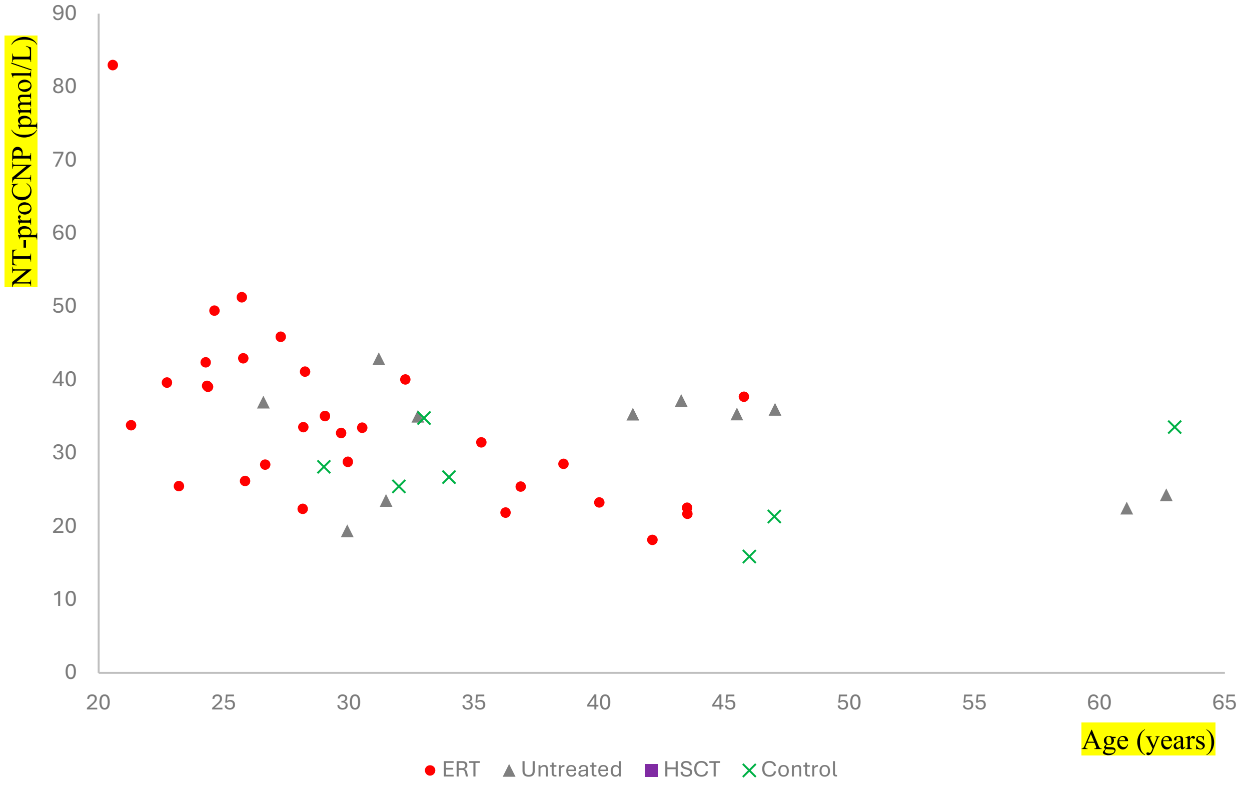

Figure S1. NT-proCNP levels in plasma from MPS IVA patients and healthy controls.

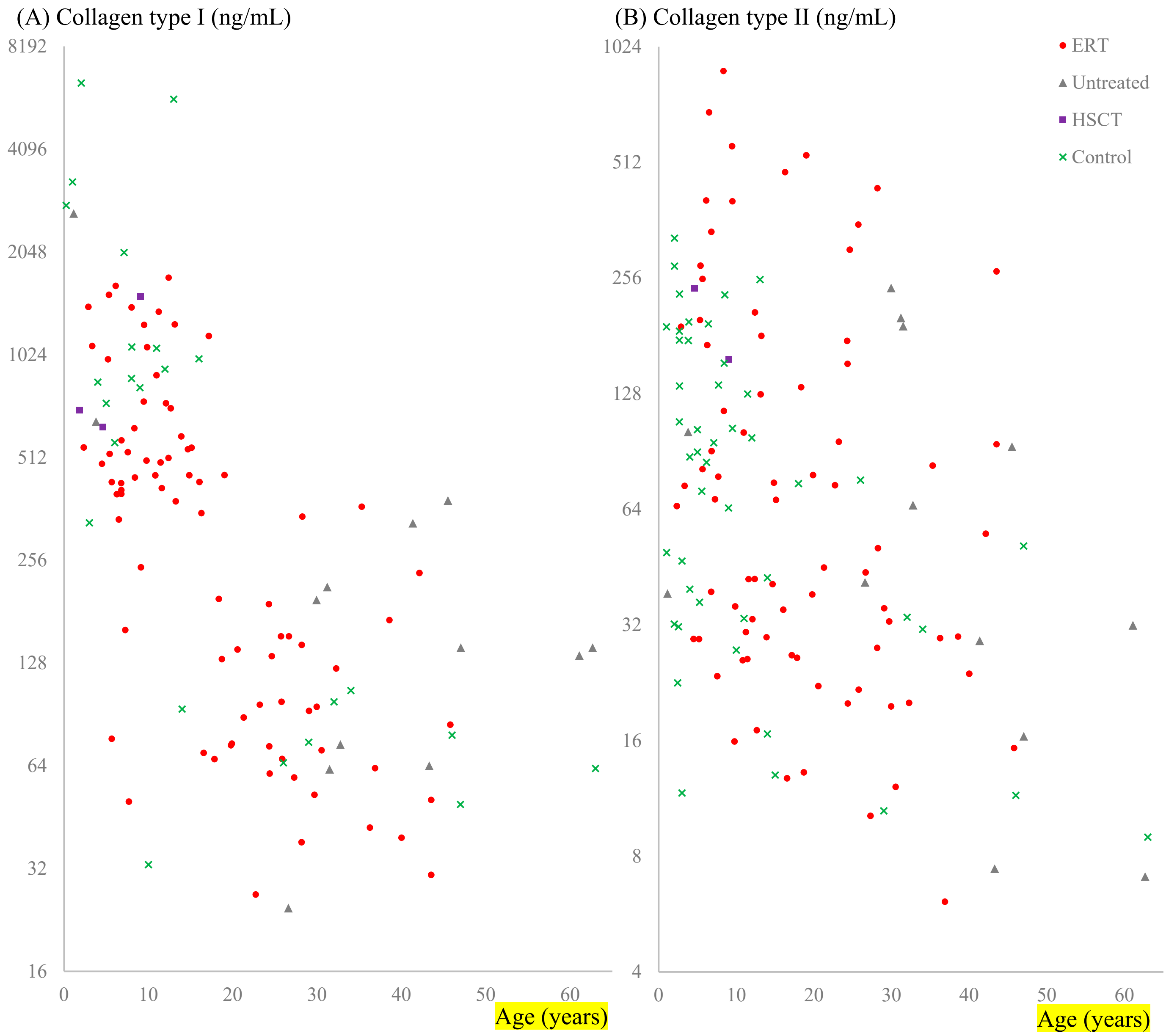

Figure S2. **Collagen type I (A) and II (B) levels in patients and controls.**

Both vertical axes are presented on a base-2 logarithmic scale.

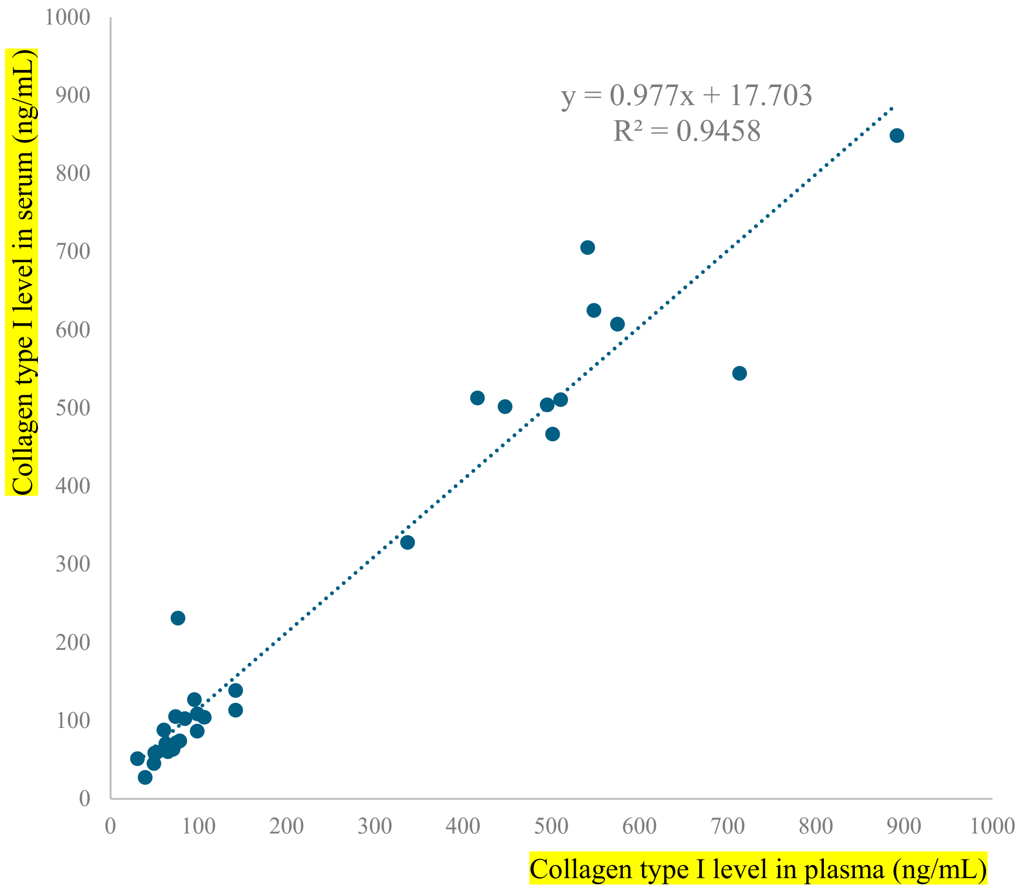

Figure S3. Collagen type I levels, difference in serum or plasma.

Each dot represents the concentration of collagen type I in plasma (horizontal axis) and serum (vertical axis) collected simultaneously from the same patient or healthy control.

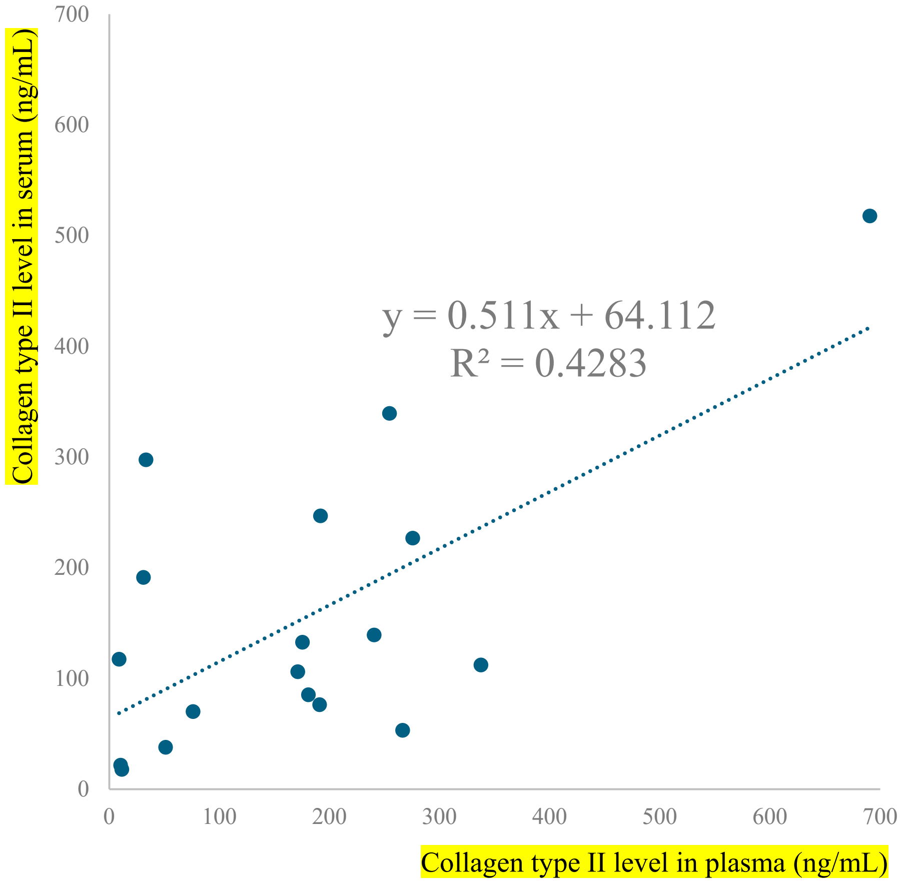

Figure S4. Collagen type II levels, difference in serum or plasma.

Each dot represents the concentration of collagen type II in plasma (horizontal axis) and serum (vertical axis) collected simultaneously from the same patient or healthy control.

Table S2: Correlation between NT-proCNP and other biomarkers in pediatric age groups

Plasma C6S	r or ρ	p	number of patients
≤ 8 y	r = -0.043	0.8543	21
>8, ≤ 13 y	r = 0.116	0.6682	16
>13, ≤ 20 y	r = 0.503	**0.0472**	16
Urinary C6S			
≤ 8 y	r = 0.365	0.1501	17
>8, ≤ 13 y	r = 0.425	0.1011	16
>13, ≤ 20 y	r = 0.634	**0.0084**	16
Plasma mono-sulfated KS			
≤ 8 y	r = -0.056	0.8048	22
>8, ≤ 13 y	r = 0.176	0.4986	17
>13, ≤ 20 y	r = 0.387	0.1382	16
Urinary mono-sulfated KS			
≤ 8 y	ρ = 0.176	0.4981	17
>8, ≤ 13 y	ρ = 0.441	0.0872	16
>13, ≤ 20 y	ρ = 0.424	0.1021	16
Plasma di-sulfated KS			
≤ 8 y	r = -0.075	0.7406	22
>8, ≤ 13 y	r = -0.080	0.7615	17
>13, ≤ 20 y	r = 0.363	0.1672	16
Urinary di-sulfated KS			
≤ 8 y	ρ = 0.0343	0.8960	17
>8, ≤ 13 y	ρ = 0.438	0.0895	16
>13, ≤ 20 y	ρ = 0.262	0.3274	16
Plasma KS ratio			
≤ 8 y	r = -0.095	0.6736	22
>8, ≤ 13 y	r = -0.179	0.4913	17
>13, ≤ 20 y	r = 0.007	0.9803	16
Urinary KS ratio			
≤ 8 y	r = -0.323	0.2054	17
>8, ≤ 13 y	r = -0.156	0.5633	16
>13, ≤ 20 y	r = -0.147	0.5861	16

r: Pearson correlation coefficient

ρ: Spearman correlation coefficient

Table S3. PCR conditions and primer pairs

**PCR 1:**			**PCR 2:**			**PCR 3:**		
93 °C	3 min	1 cycle	93 °C	3 min	1 cycle	98 °C	5 min	1 cycle
93 °C<br>70 °C	30 sec<br>30 sec	40 cycles	93 °C<br>67 °C	30 sec<br>3 min	35 cycles	98 °C<br>63 °C<br>72 °C	30 sec<br>30 sec<br>1 min	35 cycles
72 °C	10 min	1 cycle	72 °C	10 min	1 cycle	72 °C	5 min	1 cycle

Primer sequence			Amplified exons	Nucleotide length of amplified DNA	PCR condition
5'	CTGGCTCAGGCCCCGCCCCACT	3'	Exon 1	393 base pairs	PCR 1
5'	CTTCCCCCACCTCGCTCCTCCCTCCAT	3'			
5'	CTGGCTTCCACGGTCCCCGACAC	3'	Exon 2, 3, 4	2050 base pairs	PCR 2
5'	CCCAAGACACCCTCCTCATTTGGAAACT	3'			
5'	CTCAGTGCTGGAGGGTGCTCGTCTTA	3'	Exon 5, 6, 7, 8	2822 base pairs	PCR 2
5'	AGGCTCGGTGACATCTGCTCCTCC	3'			
5'	CCCTTTGTCCCTATGACCAGTCTCAGT	3'	Exon 9	261 base pairs	PCR 3
5'	GGGATGGCTGCAGGCCTGGACCT	3'			
5'	TGTGAGCATGTATGCATATCTGTAGACCCA	3'	Exon 10	490 base pairs	PCR 3
5'	AAGCCAGGGCCTCGCTGTGCTT	3'			
5'	TGATGACAGACGCAGCCCAGAGCC	3'	Exon 11, 12	2511 base pairs	PCR 2
5'	GCACGTGTGGGTATGAATAGCAACAGCA	3'			
5'	TGCTCACTGTGGTTCTCAGCCCGTT	3'	Exon 13, 14	3926 base pairs	PCR 2
5'	GTCTGCAGGTGCTGTCTGTCTGGCTT	3'			
