# Supplementary material for: Identification of Surrogate Biomarkers for Mucopolysaccharidosis Type IVA
Source: Int J Mol Sci. 2025 May 21;26(10):4940. doi: 10.3390/ijms26104940 (PMC12112068; doi:10.3390/ijms26104940)
Supplement: Supplementary file 1 [file ijms-26-04940-s001.zip › ijms-3624959-Table S1.pdf]

Patient ID	Sex	Phenotype	Race	Height (Z-score)	Height (cm)	Growth velocity (cm/year)	Age (year)	NT-proCNP		col I		col II		C6S		KS		Di S KS		KS ratio		Genotype		Treatment
								plasma (pmol/L)	serum (pmol/L)	plasma (ng/mL)	serum (ng/mL)	plasma (ng/mL)	serum (ng/mL)	Plasma (ng/mL)	Urine (ng/mg cre)	Plasma (ng/mL)	Urine (ng/mg cre)	Plasma (ng/mL)	Urine (ng/mg cre)	Plasma	Urine	Allele 1	Allele 2	
M01	F	Severe	White or Caucasian	-6.34	121.9	-1.7454	17.16	66.96		1161.61		26.71		3.619	650.93	376.27	1791.5	105.34	1498.2	0.2187	0.4554	c.740G>A (G247D)	c.901G>T (G301C)	ERT
				-6.73	119.2		18.70	45.41		131.47		13.23		0.566	200.19	523.72	804.4	131.69	1576.1	0.2009	0.6621			
M02	F	Severe	Chinese		101.5		38.57	29.90		171.05		29.85		1.430	96.00	344.00	1421.0	166.00	1510.0	0.3252	0.5151	c.953T>G (M318R)	c.1567T>G (X523EextX93)	ERT
							40.00	23.28		39.44	27.17	23.89		0.147	255.99	455.91	1199.2	162.02	2347.0	0.2622	0.6618			
M03	F	Severe	White or Caucasian		99.2		24.62	52.66		134.07		303.20		1.230	171.00	700.00	3390.0	256.00	3518.0	0.2676	0.5092	c.448delC (H150Tfs*3)	c.651_652insG (K218Efs*45)	ERT
M04	F	Severe	White or Caucasian		97.7		42.12	20.04		235.05		55.29		0.770	120.00	396.00	2979.0	163.00	2187.0	0.2912	0.4234	c.675dupC (F226Lfs*37)	unknown	ERT
							43.51	22.56		30.68	51.13	266.49	53.20	0.770	159.44	602.89	1314.2	231.89	3178.0	0.2778	0.7075			
M05	F	Severe	White or Caucasian	-9.93	98	0.4182	18.35	91.70		197.36		133.08		3.620	651.00	376.00	1791.0	105.00	1498.0	0.2187	0.4554	c.346G>A (G116S)	c.1156C>T (R386C)	ERT
				-9.76	98.6		19.78	64.75		73.52		38.45		1.851	327.07	662.02	2415.1	223.13	4965.3	0.2521	0.6728			
M06	F	Severe	White or Caucasian	-8.52	107		19.89	41.14		74.34		78.61		1.590	239.00	408.00	5209.0	182.00	6390.0	0.3086	0.5509	c.346G>A (G116S)	c.1156C>T (R386C)	ERT
							21.29	33.83		88.69		45.15		0.799	360.88	473.65	1873.7	177.88	4180.8	0.2730	0.6905			
M07	M	Severe	White or Caucasian		124		32.25	41.50		123.40		20.07		1.810	194.00	309.00	1417.0	155.00	2037.0	0.3338	0.5898	c.337A>T (I113F)	c.1171A>G (M391V)	No ERT for 2 years and 8 months
M08	M	Severe	White or Caucasian	no record	no record		8.00	no data		1408.36		no data		19.608	no data	1377.86	no data	674.28	no data	0.3286	no data	c.122T>A (M41K)	c.122T>A (M41K)	ERT
				-6.09	99	-1.0046	9.03	91.53		1514.31		157.13		0.720	261.00	1196.00	12063.0	418.00	10503.0	0.2588	0.4654			Received HSCT 1 year ago
				-6.91	97.6		10.42	no data		no data		no data		no data	no data	no data	no data	no data	no data	no data	no data			
M10	M	Attenuated	White or Caucasian	1.22	151.3	4.9689	10.82	21.86		454.06		25.90		1.200	146.85	601.44	146.8	212.00	2743.6	0.2606	0.9492	c.338T>C (I113T)	c.1219A>C (N407H)	ERT
				1.01	159		12.37	157.56		510.50	510.48	42.15		1.167	290.79	1202.76	1280.5	397.29	2220.8	0.2483	0.6343			
M11	F	Attenuated	White or Caucasian	-5.36	127.6	-0.3472	15.11	53.59		547.22		67.72		1.550	159.00	435.00	2997.0	156.00	2883.0	0.2640	0.4902	c.421T>A (W141R)	unknown	ERT
				-5.53	127.1	-0.7717	16.55	36.41		69.83		12.77		0.941	267.34	579.80	2171.4	138.39	3669.9	0.1927	0.6283			
				-5.63	126.5		17.33	no data		no data		no data		no data	no data	no data	no data	no data	no data	no data	no data			
M12	F	Severe	White or Caucasian		104.3		26.65	32.13		153.27		43.86		0.820	356.00	490.56	4315.0	163.00	5356.0	0.2494	0.5538	c.697G>A (D233N)	c.1034T>C (L345P)	No ERT for 4 years and 4 months
							28.15	22.42		38.26		27.90		0.554	214.61	678.66	3824.6	170.09	8035.8	0.2004	0.6775			
M13	M	Severe	Black or African American	-7.52	99	0.7024	13.13	67.21		1256.18		127.52		1.000	328.76	1088.00	9675.0	336.00	9676.0	0.2360	0.5000	c.251C>A (A84E)	c.319G>A (A107T)	ERT
				-6.75	100.2		14.84	57.93		454.51		75.08		0.562	579.41	1438.80	3818.2	510.78	6682.3	0.2620	0.6364			
M14	F	Attenuated	White or Caucasian	-5.7	126	0.0000	16.29	53.30		351.67		482.73		1.138	227.11	404.34	1496.2	151.37	1873.4	0.2724	0.5560	c.740G>A (G247D)	unknown	ERT
				-5.7	126		17.83	35.52		67.02		26.29		0.305	10.45	551.45	1923.7	228.40	3700.5	0.2929	0.6580			
M15	M	Severe	White or Caucasian	-2.39	99.5	0.6702	5.32	85.15		1534.94		199.04		1.406	1680.65	1378.06	21140.8	578.00	30952.0	0.2955	0.5942	c.498delC (F167Lfs*32)	c.901G>T (G301C)	ERT
				-3.84	100.5		6.81	103.81		575.01	607.13	90.70		0.353	826.35	2078.56	8782.9	878.69	21694.6	0.2971	0.7118			
M16	M	Severe	Filipino	-5.09	85.6	0.5907	5.21	83.29		993.08		29.41		1.760	1050.00	1305.00	20783.0	509.00	30026.0	0.2806	0.5910	c.228C>A (N76K)	c.1480A>G (p.M494V)	ERT
				-5.95	86.2		6.23	no data		no data		no data		no data	no data	no data	no data	no data	no data	no data	no data			
				no record	no record		6.77	99.67		430.96		39.04		1.708	1025.54	1818.31	7679.1	690.47	17611.3	0.2752	0.6964			
M19	F	Severe	Black or African American	-6.49	98.5	4.0803	9.44	167.19		746.62		564.05		0.952	167.56	1081.37	6615.5	363.01	7329.8	0.2513	0.5256	c.245C>T (S82L)	unknown	ERT
				-5.64	104.7		10.96	168.59		891.95	848.34	101.44		0.738	475.45	1415.26	4146.3	411.37	9156.9	0.2252	0.6883			
M20	F	Attenuated	White or Caucasian	-4.09	111.1	8.6044	9.49	41.26		1252.63		405.41		1.709	160.67	1005.81	6479.6	353.71	7415.8	0.2602	0.5337	c.498delC (F167Lfs*32)	c.1474G>A (A492T)	ERT
				-3.53	116		10.06	no data		no data		no data		no data	223.35	no data	2902.7	no data	6025.5	no data	0.6749			
M21	M	Severe	White or Caucasian	-4.08	95	1.8123	6.12	109.48		1630.22		407.38		0.664	999.52	1328.62	16375.5	496.00	21635.0	0.2718	0.5692	c.651_652insG (K218Efs*45)	c.1159G>A (G387S)	ERT
				-5.22	97.6		7.55	90.06		530.66		23.54		0.940	290.59	1945.86	13424.1	834.94	32473.5	0.3003	0.7075			
M22	M	Severe	White or Caucasian	-5.45	100	-3.7640	8.34	98.16		623.71		885.55		1.939	1117.00	1746.74	8848.0	626.62	10450.0	0.2640	0.5415	c.651_652insG (K218Efs*45)	c.1159G>A (G387S)	ERT
				-7.24	94.6		9.78	79.68		501.46	466.52	15.93		0.488	172.32	1859.69	13775.8	579.14	35514.4	0.2375	0.7205			
M25	M	Severe	White or Caucasian		98.5		41.34	34.96		328.91		29.11		1.300	358.00	521.00	5137.0	183.00	5402.0	0.2597	0.5126	c.155C>T (P52L)	c.337A>T (I113F)	Never received ERT
							43.28	37.18	36.84	64.04		7.43		0.789	265.10	700.71	3393.1	375.49	6966.1	0.3489	0.6725			
M26	M	Severe	Filipino	-7.03	103	1.2015	12.39	82.04		1721.96		208.32		1.245	432.45	1149.91	9221.1	473.17	12986.3	0.2915	0.5848	c.93delC (N32Tfs*97)	c.946G>A (G316R)	ERT
				-6.61	104.5		13.64	no data		no data		no data		no data	no data	no data	no data	no data	no data	no data	no data			
				no record	no record		13.89	93.06		590.44		29.73		0.860	665.80	1429.69	8832.3	641.40	18791.5	0.3097	0.6803			
M28	F	Severe	Mixed		108		31.20	39.16		213.71		201.71		1.110	262.00	397.00	5047.0	150.00	7971.0	0.2744	0.6123	c.868G>A (G290S)	c.868G>A (G290S)	Never received ERT
			Japanese and Caucasian				32.75	35.03		73.89	105.15	65.66		0.525	no data	572.25	no data	247.40	no data	0.3018	no data			
M30	F	Severe	White or Caucasian		111.6		43.52	19.27		50.87		94.51		0.980	67.00	256.00	1841.0	154.00	2094.0	0.3762	0.5322	c.121-12T>C	c.121-12T>C	ERT
M31	M	Attenuated	Black or African American	-1.29	129.5	5.2540	9.86	49.42		1077.19		35.78		1.335	255.72	1359.45	4043.1	445.86	3538.2	0.2470	0.4667	c.935C>G (T312S)	c.1520G>T (C507F)	ERT
				-1.11	137.8		11.44	28.52		495.35	503.91	26.08		0.694	353.80	1760.67	2116.2	808.48	4436.4	0.3147	0.6770			
M32	F	Severe	White or Caucasian		97		45.50	30.75		382.95		93.22		1.530	303.00	384.00	2473.0	205.00	4059.0	0.3477	0.6215	c.121-12T>C	c.121-12T>C	Never received ERT
							47.03	36.00		142.01	138.47	16.43		1.396	134.88	549.87	2197.8	274.49	4776.7	0.3330	0.6849			
M33	F	Severe	White or Caucasian		118.2		29.94	18.67		195.93		241.23		0.470	207.00	249.00	4071.0	151.00	7125.0	0.3776	0.6364	c.1012C>T (Q338X)	c.1171A>G (M391V)	Never received ERT
							31.48	23.55		62.53	62.52	191.96	246.86	0.481	187.90	418.14	2311.4	263.65	5667.5	0.3867	0.7103			
M34	M	Severe	White or Caucasian		112.2		28.17	32.81		144.74		438.58		0.660	536.00	299.00	6073.0	140.00	11108.0	0.3192	0.6465	c.121A>T (M41L)	c.121A>T (M41L)	ERT
							29.69	32.76		52.67	59.24	32.68		0.548	379.95	505.03	1050.8	319.48	3224.2	0.3875	0.7542			
M35	M	Severe	White or Caucasian	-6.86	100.3	-0.9167	11.23	132.07		1368.54		30.66		1.279	492.55	1292.08	44082.3	438.00	37784.4	0.2532	0.4615	c.139G>A (G47R)	c.1156C>T (R386C)	No ERT for 4 years
				-7.69	99		12.64	208.20		713.59	544.15	17.03		1.811	886.16	1472.18	20231.4	437.43	43131.6	0.2291	0.6807			
M36	F	Severe	Asian Indian		103.8		25.72	51.52		153.27		352.69		1.620	155.00	401.00	3026.0	149.00	3274.0	0.2716	0.5197	c.346G>A (G116S)	c.346G>A (G116S)	ERT
							27.27	45.89		59.18		10.20		0.488	136.93	491.52	1863.2	199.36	3531.4	0.2886	0.6546			
M37	M	Severe	Asian Indian	-10.12	100		19.01	80.42		454.78		534.18		1.917	145.00	534.96	3716.2	164.46	3690.8	0.2351	0.4983	c.346G>A (G116S)	c.346G>A (G116S)	ERT
							20.56	82.99		140.30		22.18		0.945	173.48	548.48	1590.7	200.59	2989.1	0.2678	0.6527			
M38	F	Attenuated	White or Caucasian	-5.63	126.4		16.04	40.37		434.15		35.07		1.097	303.62	450.29	1428.6	241.43	2255.3	0.3490	0.6122	c.1559G>A (W520X)	unknown	ERT
M39	F	Attenuated	White or Caucasian	-3.54	125		12.08	74.16		737.98		33.15		1.236	253.50	693.05	4176.3	331.92	4364.6	0.3238	0.5110	c.1559G>A (W520X)	unknown	ERT
M41	M	Severe	White or Caucasian		116		35.29	30.99		367.31		83.17		1.150	162.00	310.00	1289.0	206.00	1953.0	0.3992	0.6024	c.121A>T (M41L)	c.498delC (F167Lfs*32)	ERT
							36.86	25.45		63.03	67.32	6.10		0.658	195.98	579.80	868.3	366.58	2456.6	0.3873	0.7388			
M42	F	Severe	White or Caucasian		107.4		28.24	40.01		343.84		50.72		0.979	168.47	601.32	1138.1	219.99	1977.7	0.2679	0.6347	c.860C>T (S287L)	c.1055T>C (L352P)	ERT
							29.95	28.83	30.45	95.32	126.84	19.64		0.508	302.95	401.10	965.0	307.37	2507.2	0.4339	0.7221			
M43	M	Severe	White or Caucasian		105.5		29.04	32.97	42.59	92.74		35.38		0.480	319.75	685.04	2259.7	346.97	5033.7	0.3362	0.6902	c.121A>T (M41L)	c.901G>T (G301C)	ERT
							30.52	33.48	38.36	71.11	63.29	12.13		0.692	325.21	734.67	1882.7	406.11	4136.5	0.3560	0.6872			
M44	F	Severe	White or Caucasian		124.3		24.28	42.42	52.39	190.51		175.59	132.67	0.687	41.41	558.41	625.4	364.04	1181.5	0.3946	0.6539	c.1171A>G (p.M391V)	c.502G>A (p.G168R)	ERT
							25.85	26.22	28.67	67.07		no data		no data	no data	466.39	no data	68.13	no data	0.1275	no data			
M45	F	Attenuated	White or Caucasian		139.7		61.08	21.34	23.87	134.63		31.96		1.901	193.33	674.62	1328.8	302.20	2696.3	0.3094	0.6699	c.740G>A (G247D)	c.761A>G (Y254C)	Never received ERT
							62.66	24.33	23.53	142.01	113.22	7.09		1.156	228.59	474.64	1481.0	323.05	4151.2	0.4050	0.7370			
M46	F	Severe	White or Caucasian	no record	no record		6.92	no data		no data		no data		no data	536.88	no data	6476.0	no data	12381.9	no data	0.6566	c.167C>A (T56N)	c.502G>A (G168R)	ERT
				-5.54	100.6		8.40	97.35	102.91	447.46	501.52	115.41		1.454	467.63	1470.70	3245.9	723.33	6845.8	0.3297	0.6784			
M47	F	Severe	Some other race		99.7		24.33	39.20	44.56	73.05		153.16		0.270	125.59	633.03	762.7	295.98	1434.6	0.3186	0.6529	c.139G>A (G47R)	c.466T>C (F156L)	ERT
							25.78	42.97	43.23	98.59	108.43	21.73		0.953	280.61	442.22	1144.1	296.22	3456.6	0.4011	0.7513			
M48	F	Severe	White or Caucasian	-7.11	108.3	2.9054	13.23	67.76	79.92	381.24		180.95	85.29	0.720	444.66	1510.31	2630.6	837.58	5419.5	0.3567	0.6732	c.139G>A (G47R)	c.466T>C (F156L)	ERT
				-7.66	112.5		14.68	78.33	78.18	541.20	705.20	40.87		2.101	1091.70	1091.25	5070.4	535.79	13697.7	0.3293	0.7298			
M49	M	Severe	White or Caucasian	-6.44	92	-0.4231	7.68	60.10		50.29	58.30	77.85		0.700	760.26	1148.62	9443.7	289.49	22815.5	0.2013	0.7073	c.1156C>T (R386C)	c.1156C>T (R386C)	ERT
				-7.63	91.4		9.10	no data		no data		no data		no data	no data	no data	no data	no data	no data	no data	no data			
M50	F	Severe	White or Caucasian	-3.33	97	1.4287	5.65	76.66	113.12	433.89		81.45		1.228	973.31	2134.76	10126.8	949.80	19754.3	0.3079	0.6611	c.451C>A (P151T)	c.477G>A (W159X)	ERT
				-4.81	99.3		7.26	59.80	62.14	160.01		67.95		0.537	470.88	1079.44	5293.2	627.46	15186.4	0.3676	0.7415			
M51	M	Attenuated	White or Caucasian	-0.9	91.5	7.3423	2.89	117.43	123.86	1413.43		191.15	76.21	1.274	1050.19	1977.91	9657.5	1005.90	17357.4	0.3371	0.6425	c.451C>A (P151T)	c.477G>A (W159X)	ERT
				-0.52	103.3		4.50	90.39	99.20	490.66		29.44		0.851	362.23	1643.89	7101.5	1098.45	19298.8	0.4006	0.7310			
M52	F	Attenuated	Multiple Races	no record	no record		2.50	no data		704.57		no data		8.600	no data	1277.00	no data	739.00	no data	0.3668	no data	c.651_652insG (K218Efs*45)	unknown	Received HSCT 11 months ago
				no record	no record		4.60	64.94	69.63	629.47		240.58	139.25	0.969	no data	2864.17	no data	1297.21	no data	0.3117	no data			
				-2.54	104		6.21	no data		no data		no data		no data	282.91	no data	3564.3	no data	9501.5	no data	0.7272			
M53	F	Attenuated	Chinese		127		23.20	25.51	26.75	96.69		95.94		0.344	631.73	493.47	3456.4	312.09	8834.6	0.3874	0.7188	c.1482+5G>C	c.1498G>T (G500C)	No ERT for 13 months
							24.37	39.07		60.76	87.76	20.00		0.521	347.40	477.48	1064.4	297.61	2491.3	0.3840	0.7007			ERT
M54	F	Attenuated	White or Caucasian		127.7		36.26	21.91	24.33	42.22		29.57		0.030	257.93	354.45	1654.7	232.33	3417.2	0.3959	0.6738	not tested	not tested	No ERT for 5 years and 9 months
M55	F	Severe	White or Caucasian	-3.04	96.7	2.2784	5.39	60.13		524.53		275.69	226.74	0.820	1101.96	1426.93	6701.4	996.57	16964.0	0.4112	0.7168	c.1339G>C (D447H)	unknown	ERT
				-4.2	99.9		6.80	104.42	114.22	400.51		no data		no data	no data	1309.95	no data	182.19	no data	0.1221	no data			
M56	F	Boundary	White or Caucasian	-1.44	93.6		3.79	68.65	73.56	652.30		101.82		0.805	769.50	1415.58	10847.2	774.82	23588.0	0.3537	0.6850	c.740G>A (G247D)	c.1451C>A (P484H)	Never received ERT
M57	M	Undefined	White or Caucasian	0.41	78.6		1.13	99.12	123.97	2654.07		38.64		0.438	no data	1233.38	no data	524.20	no data	0.2982	no data	c.740G>A(G247D)	c.1451C>A (P484H)	Never received ERT
M58	M	Severe	White or Caucasian	-4.38	97.2	1.1454	6.78	78.31	80.63	410.94		337.53	112.11	1.583	754.26	1794.20	10624.4	1136.94	20107.1	0.3879	0.6543	c.1012C>T (Q338X)	c.181C>G (R61G)	ERT
				-5.64	99		8.36	no data		no data		no data		no data	no data	no data	no data	no data	no data	no data	no data			
M59	M	Severe	White or Caucasian	-4.14	95.6	0.8696	6.26	70.13		399.99		171.26	106.08	0.691	508.39	1580.81	5719.4	938.12	12450.1	0.3724	0.6852	c.337A>T (p.I113F)	c.901G>T (p.G301C)	ERT
				-5.59	97		7.87	no data		no data		no data		no data	no data	no data	no data	no data	no data	no data	no data			
M60	F	Severe	Some other race		100.1		26.58	36.97		24.54		41.30		0.614	561.09	638.58	4555.4	182.48	6846.3	0.2222	0.6005	c.901G>T (G301C)	c.1156C>T (R386C)	Never received ERT
M63	F	Severe	White or Caucasian		101.6		22.72	39.65		26.87		73.96		0.883	35.51	599.60	1555.7	143.06	2671.3	0.1926	0.6320	c.331C>T (Q111X)	c.1365-2A>G	ERT
M64	M	Severe	Black or African American	-3.74	94.5	2.5858	5.64	72.85		76.81	231.08	254.57	339.38	1.444	467.21	2255.93	9498.9	510.41	22129.9	0.1845	0.6997	c.633+1G>C	c.1558T>C (W520R)	ERT
				-4.59	98.5		7.19	no data		no data		no data		no data	no data	no data	no data	no data	no data	no data	no data			
M65	M	Severe	Black or African American	-2.9	103.5	2.5858	6.50	46.00		336.98	327.76	690.76	517.61	1.259	226.07	1868.31	17080.5	444.92	31895.5	0.1923	0.6512	c.633+1G>C	c.1558T>C (W520R)	ERT
				-3.78	107.5		8.04	no data		no data		no data		no data	no data	no data	no data	no data	no data	no data	no data			
M66	F	Severe	White or Caucasian	-2.1	87.9	4.9677	3.34	72.27		1086.26		73.67		1.522	no data	2223.83	no data	805.04	no data	0.2658	no data	c.1156C>T (R386C)	unknown	ERT
				-2.5	94.85		4.74	no data		no data		no data		no data	no data	no data	no data	no data	no data	no data	no data			
M67	M	Attenuated	White or Caucasian	-1.55	135.6		11.58	55.82		416.33	512.76	42.12		1.021	306.23	1447.90	3819.0	754.75	8028.3	0.3427	0.6776	c.1171 A>G (M391V)	c.331C>T (Q111X)	ERT
M68	M	Attenuated	White or Caucasian		152.4		45.78	37.71	27.05	84.48	102.37	15.31		1.055	296.78	325.10	570.1	248.50	1515.9	0.4332	0.7267	c.1156C>T (R386C)	c.181C>T (R61W)	ERT
M69	M	Severe	Asian	-3.03	79.5		2.33	66.48		548.24	624.57	65.27		1.331	no data	1801.51	no data	1038.02	no data	0.3656	no data	c.106_111del (p.L36_L37del)	c.1201C>T (H401Y)	ERT
M71	M	Attenuated	White or Caucasian	-3	116.1		9.12	59.50	54.92	244.27		no data		no data	no data	967.56	no data	89.71	no data	0.0849	no data	c.1219A>C (N407H)	unknown	ERT
Adult controls																								
	M		Asian Indian				26	31.97	28.00	65.31	60.00	76.23	70.13											
	F		South Asian				29	28.14	26.62	75.05	71.67	10.51	21.75											
	F		White or Caucasian				32	25.47	24.62	98.51	86.43	33.51	297.45											
	F		White or Caucasian				34	26.74	30.86	106.34	104.20	31.20	191.33											
	F		East Asian				46	15.89	15.46	78.74	73.82	11.53	17.95											
	M		East Asian				47	19.64	20.08	49.34	45.02	51.36	37.82											
	M		East Asian				63	33.56	38.82	62.88	70.18	8.98	117.29											
